# Anti-Vasculogenic, Antioxidant, and Anti-Inflammatory Activities of Sulfated Polysaccharide Derived from *Codium tomentosum*: Pharmacokinetic Assay

**DOI:** 10.3390/ph17060672

**Published:** 2024-05-23

**Authors:** Marwa Lakhrem, Malek Eleroui, Zakaria Boujhoud, Amal Feki, Amel Dghim, Sanah Essayagh, Said Hilali, Marwa Bouhamed, Choumous Kallel, Nathalie Deschamps, Bertrand de Toffol, Jean Marc Pujo, Riadh Badraoui, Hatem Kallel, Ibtissem Ben Amara

**Affiliations:** 1 Laboratory of Medicinal and Environment Chemistry, Higher Institute of Biotechnology, University of Sfax, Sfax 3000, Tunisia; lakhremma@gmail.com (M.L.); aroui.malek@gmail.com (M.E.); amal.feki05@gmail.com (A.F.); dsirine51@gmail.com (A.D.); 2 Laboratory of Health Sciences and Technologies, Higher Institute of Health Sciences of Settat, Settat 26000, Morocco; z.boujhoud@uhp.ac.ma; 3 Laboratory Agrifood and Health, Faculty of Science and Technology, Hasan First University of Settat, Settat 26000, Morocco; essayagh@gmail.com (S.E.); said.hilali@gmail.com (S.H.); 4 Laboratory of Anatomopathology, CHU Habib Bourguiba, University of Sfax, Sfax 3029, Tunisia; marwa4272@gmail.com; 5 Laboratory of Hematology, CHU Habib Bourguiba, University of Sfax, Sfax 3029, Tunisia; choumous.kallel@gmail.com; 6 Neurology Department, Cayenne General Hospital, Cayenne 97300, French Guiana; nathalie.deschamps@ch-cayenne.fr (N.D.); bertrand.detoffol@ch-cayenne.fr (B.d.T.); 7 Clinical Investigation Center, CIC INSERM 142, Cayenne General Hospital Andrée Rosemon, Guiana University, Cayenne 97300, French Guiana; 8 Emergency Department, Cayenne General Hospital, Cayenne 97300, French Guiana; tamac1966@gmail.com; 9 Department of Biology, University of Ha’il, Ha’il 81451, Saudi Arabia; riadh.badraoui@fmt.utm.tn; 10 Section of Histology-Cytology, Medicine Faculty of Tunis, University of Tunis El Manar, La Rabta 1007, Tunisia; 11 Biome and Immunopathology CNRS UMR-9017, Inserm U 1019, Université de Guyane, Cayenne 97300, French Guiana; kallelhat@gmail.com; 12 Intensive Care Unit, Cayenne General Hospital, Cayenne 97300, French Guiana

**Keywords:** polysaccharide, antioxidant activities, oxidative stress, inflammatory reaction, *Codium tomentosum*, pharmacokinetics, bioavailability, anti-angiogenic action

## Abstract

The purpose of this paper was to investigate the anti-inflammatory and anti-angiogenic activities of sulfated polysaccharide from *C. tomentosum* (PCT) using carrageenan (CARR)-induced paw edema in a rat model and anti-vasculogenic activity on a chorioallantoic membrane assay (CAM) model. Based on in vitro tests of anti-radical, total antioxidant, and reducing power activities, PCT presents a real interest via its antioxidant activity and ability to scavenge radical species. The in vivo pharmacological tests suggest that PCT possesses anti-inflammatory action by reducing paw edema and leukocyte migration, maintaining the redox equilibrium, and stabilizing the cellular level of several pro-/antioxidant system markers. It could significantly decrease the malondialdehyde levels and increase superoxide dismutase, glutathione peroxidase, and glutathione activities in local paw edema and erythrocytes during the acute inflammatory reaction of CARR. PCT pretreatment was effective against DNA alterations in the blood lymphocytes of inflamed rats and reduced the hematological alteration by restoring blood parameters to normal levels. The anti-angiogenic activity results revealed that CAM neovascularization, defined as the formation of new vessel numbers and branching patterns, was decreased by PCT in a dose-dependent manner, which supported the in silico bioavailability and pharmacokinetic findings. These results indicated the therapeutic effects of polysaccharides from *C. tomentosum* and their possible use as anti-proliferative molecules based on their antioxidant, anti-inflammatory, and anti-angiogenic activities.

## 1. Introduction 

According to recent findings from various studies, angiogenesis and inflammation play a vital role in the initiation and progression of multiple diseases [1]. Nearly 50 years ago, Ref. [2] proposed the first studies on angiogenesis as a process of forming a new network of vessels from the preexisting vessels. It is a complex endothelial cell proliferation, invasion, migration, and tube formation process [3]. It is involved in developing many physiological processes like wound healing, placenta formation, embryonic development, menstrual cycle, and hair growth [4,5]. However, abnormal angiogenesis may be associated with numerous human diseases like cancer. To fulfill a solid tumor’s growing demands for oxygen, nutrients, and the excretion of metabolic waste, the creation of other blood vessels is required [3,6]. In addition, several reports demonstrated that numerous illnesses first manifest and grow due to chronic inflammatory areas [7]. In this context, the dysregulation in the inflammatory pathways induces an exaggerated and uncontrolled inflammatory response marked by excessive production of macrophages and complex interactions between resident immune cells and soluble factors followed by intense neutrophil infiltration and the release of a variety of mediators such as histamines, serotonin, bradykinins, nitric oxide, and prostaglandins [8] which causes various diseases including rheumatoid arthritis, osteoarthritis, sepsis, chronic pulmonary inflammatory disease, Crohn’s disease, ulcerative colitis, and carcinogenesis [9]. Based on these findings, suppressing blood vessel growth and inhibiting the inflammatory reaction present a new therapy option.

Numerous studies have shown that opioids and non-steroidal anti-inflammatory drugs (NSAIDs) have adverse side effects [10]. For this reason, searching for new chemical entities with higher antioxidant activities presents one of the most important challenges in food science and technology. Numerous studies support the idea that adding bioactive compounds such as phenolics, flavonoids, anthocyanins, and minerals to a diet could provide a natural treatment for various diseases, including inflammation [11].

Among bioactive metabolites, polysaccharide compounds, which are present in several algae species, have attracted more attention because of their biological activities, such as their anticoagulant [12], antitumor [13], anti-angiogenic [14], antioxidant, antiviral, anti-proliferative, and anti-inflammatory activities [12]. Additionally, polysaccharides have been evaluated in vitro and in vivo to confirm their analgesic, immunomodulatory, and anti-angiogenic effects [15,16]. Polysaccharides are able not only to prevent the migration of leukocytes to the site of inflammation [17] but also to modulate the production of numerous inflammatory factors such as cytokines, serotonin, bradykinins, nitric oxide, and interleukin implicated in the inflammatory process [18]. The scavenging and denaturing of reactive oxygen species (ROS) present an important and successful anti-inflammatory strategy that polysaccharides exert to reduce tissue damage [19]. Heterofucan, a polysaccharide from *D. menstrualis*, suppressed the inflammatory reaction by directly binding to the cell surface of leukocytes [20]. Polysaccharides extracted from *Caulerpa mexicana* and *Turbinaria ornate* have high antioxidant potential and antinociceptive/anti-inflammatory activity in mice and rats [21,22]. Therefore, the combination of algal polysaccharides’ anti-inflammatory, anti-angiogenic, and antioxidant activities can be proposed as the primary treatment strategy for several health problems correlated with the inflammatory process.

*Codium tomentosum* is green algae belonging to the family of Codiacae and is the most invasive seaweed in the world [23]. It has been widely used as a food additive and feed, particularly by populations in Southeast Asia. This species of algae is characterized by a lack of cellulose and the presence of three types of sulfated polysaccharides (arabinans, arabinogalactans, and mannans) [24]. Also, it contains other bioactive compounds such as vitamins A, C, and E, phenolics, carbohydrates, protein, chlorophylls a and b, and carotene [25].

In contrast to other Codium species, including *Codium fragile* [26], *Codium decorticatum* [27], and *Codium divaricatum* [28], *Codium tomentosum* has been the subject of very few studies focusing on its biological characteristics and biomedical potential. The exploitation of marine compounds in the medical and pharmaceutical sectors is restricted.

In this study, we concentrated for the first time on assaying the anti-inflammatory and anti-angiogenic activity of a novel polysaccharide from *C. tomentosum* (PCT). Both bioavailability and pharmacokinetic properties of the polysaccharide building blocks were assessed by in silico analyses. Then, an anti-inflammatory assessment was performed by studying the capacity of PCT to reduce hematological alteration, DNA damage, and oxidative stress on carrageenan-induced acute inflammation. Meanwhile, the anti-angiogenic activity was analyzed using the chick chorioallantoic membrane assay.

## 2. Results and Discussion

### 2.1. Extraction Yield and Chemical Analysis

Green algae are distributed extensively in many countries in the world. However, the information on their structures, applications, and biological activities is scarce compared with other algae classes (red and brown). The extraction yield of PCT was estimated to be 15.22 ± 0.05% (*w*/*w*) (Table 1) based on *C. tomentosum* dry weight. Compared with other algae species, *C. tomentosum* has an extract yield similar to *Chaetomorpha linum* (16.35 ± 0.5%) [29], but much higher than *Chondrus canaliculatus* (2.05 ± 0.5%) [30] and *Laminaria japonica* (2.3%) [31], and lower than other seaweeds like *Ulva ohnoi* (36.5 ± 3.1%), *Ulva meridionalis* (40.4 ± 3.2%), and *Monostroma latissimum* (40.4 ± 7.2%) [32]. The variation in the content of seaweed polysaccharides changes depending on species, habitat, physiological factors, season, and age [33]. For example, in the winter, Laminaria species are rich in alginic acid, and Fucus species have the highest rate of polysaccharides during autumn. The spring and summer seasons were when *Chondrus canaliculatus* biomass peaked [30]. Since this alga was harvested in the spring, the timing of the harvest might account for the observed exceptional extraction yield of *C. tomentosum*.

The chemical composition of polysaccharides was analyzed, and the results are shown in Table 1. The percentage of total sugars, evaluated by the phenol–sulfuric acid method, was 53.84% ± 0.19. This result is similar to previous studies [29,30]. Moreover, the extracts presented low protein content, accounting for 2.05% ± 0.13. The low protein concentration is predicted to correlate to the weak initial protein concentration content in the algal powder. The sulfated group was estimated at 5.71% ± 0.07. This amount seems lower than those in *Turbinaria decurrens* [34].

### 2.2. Spectroscopic Analysis of PCT

**Figure 1:**
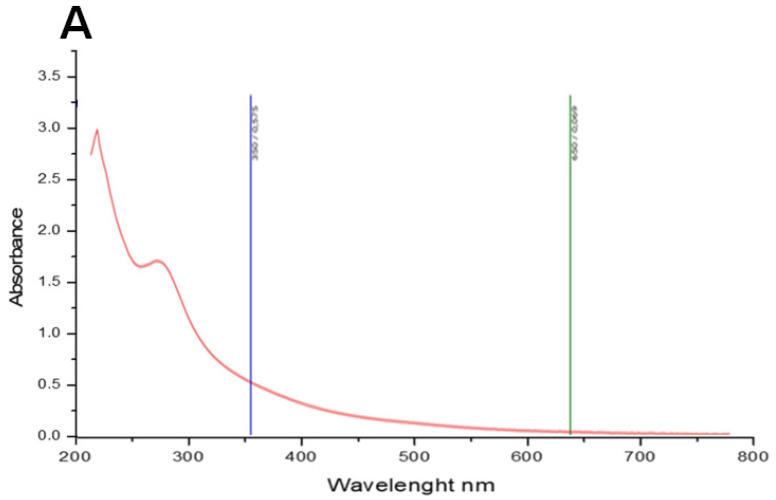

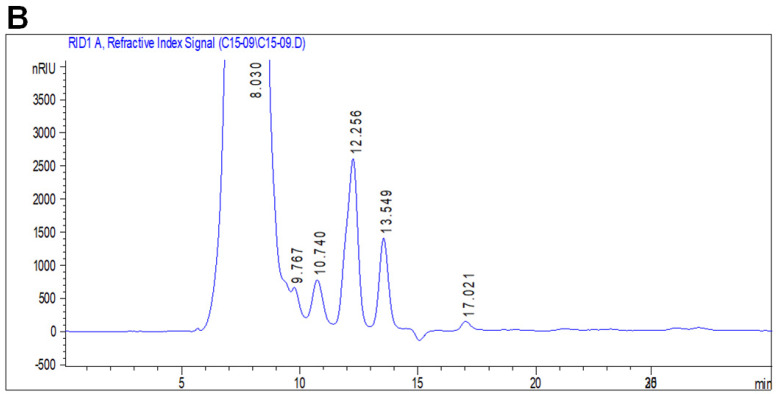
Structural analysis of polysaccharide extracted from *C. tomentosum*. (**A**) UV–visible absorption spectrum. (**B**) Monosaccharide composition analysis using HPLC-FID.

The PCT has been analyzed in the UV–visible absorption spectrum (200–800 nm). The data in Figure 1A show that PCT has maximum absorption peaks at 230 nm; subsequently, the sample was identified as a polysaccharide. Our results aligned with data recorded by [29] which presented a significantly prominent absorbance peak at 205−215 nm.

It is critical to understand the monosaccharide content of bioactive polysaccharides in research to interpret their biological activities correctly. The monosaccharide content of PCT was examined in our study using HPLC-FID, as shown in Figure 1B. Glucuronic acid, arabinose, galacturonic acid, xylose, fructose, and rhamnose are the major polysaccharide components at retention times of 8.03, 9.76, 10.74, 12.25, 13.54, and 17.02, respectively. The PCT synthesized in this study can be classified as a heteropolysaccharide due to the inclusion of several monosaccharides. Other studies prove that polysaccharides of green algae revealed complex and heterogeneous monosaccharide composition; for example, glucose, xylose, and galactose were the major monosaccharide units of polysaccharide from *Codium fragile* [35]. Polysaccharide extracted from *Codium bernabei* was composed of arabinose, fucose, xylose, mannose, galactose, and glucose [36]. This variability and diversity in polysaccharides’ composition could be explained by exogenous parameters such as temperature, light day intensity and length, and nutrient concentration in water [37].

**Figure 2:**
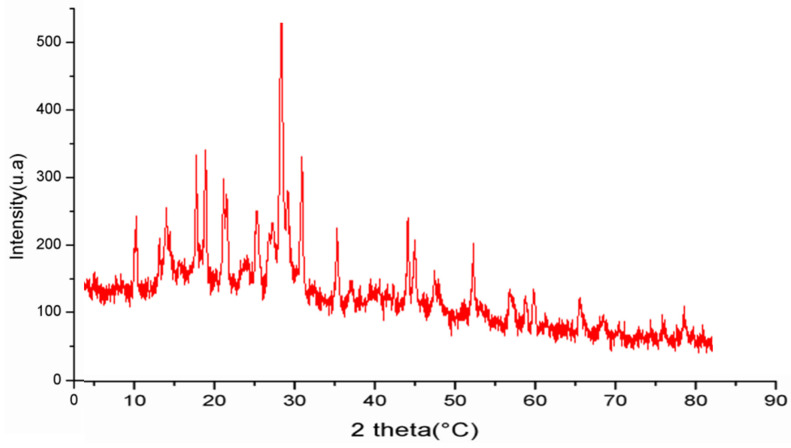
XRD spectrum of PCT.

The XRD technique was used to determine the crystalline structure of polysaccharides. The X-ray diffractogram of PCT observed between 0° and 100° is presented in Figure 2. Based on the results, PCT is a semi-crystalline polymer with one major reflection at 30°. These findings align with those found by Manikandan et al. [34]. These authors showed that polysaccharides extracted from the algae *Turbinaria decurrens* also possess a semi-crystalline structure. However, it is recognized that several properties, such as the bulk polymer’s solubility, flexibility, swelling, or opaqueness, directly impact the crystalline and semi-crystalline structure of materials.

**Figure 3:**
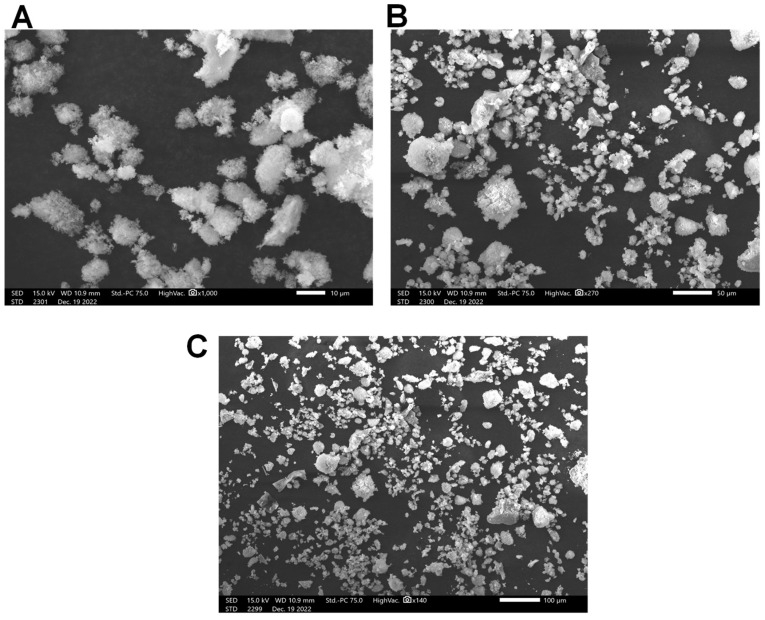
Morphological structure of PCT using scanning electron microscopy: (**A**) magnification ×10; (**B**) magnification ×50; (**C**) magnification ×100.

Scanning electron microscopy is a powerful technique used to study the surface morphology of a polymer. As shown in Figure 3, PCT comprised many variable-sized irregular fragments. The SEM images at 100-fold magnification indicated that the PCT has a granular structure with a rough surface (Figure 3C). According to the literature, the bioactivity of the polymer is probably related to its particular structure. Contrary to our results, Gao et al. [38] demonstrated that polysaccharides from *Ulva pertusa* exhibited flakes with a smooth surface.

### 2.3. Druggability, Bioavailability, and Pharmacokinetics

**Figure 4:**
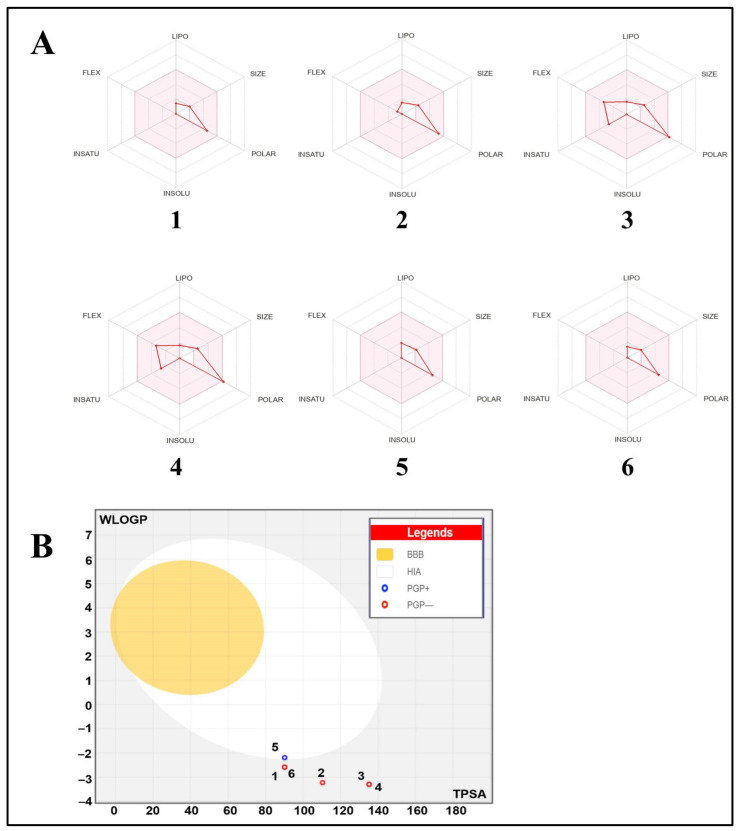
(**A**) Illustration of the oral bioavailability of PCT building blocks based on their physicochemical properties: lipophilicity (LIPO), molecular size (SIZE), polarity (POLAR), insolubility (INSOLU), insaturation (INSATU), and flexibility (FLEX). Regarding less lipophilicity, most of the studied monosaccharides and paroxetine possessed good oral bioavailability. (**B**) Illustration of the boiled egg model for the PCT monosaccharides. None of the studied PCT monosaccharides are blood–brain barrier (BBB)-permeant. Rhamnose is a P-glycoprotein substrate (PGP+) that has positive human intestinal absorption (HIA). Fucose and glucuronic acid possess the highest gastrointestinal (GI) absorption. (1) Arabinose; (2) fructose; (3) galacturonic acid; (4) glucuronic acid; (5) rhamnose; (6) xylose.

Both the druggability and pharmacokinetic properties of the PCT monosaccharides are shown in Table 2. These computational screening data of compounds are commonly assessed to avoid drug failure, specifically in advanced stages and clinical phases [39,40]. All the green-algae-identified monosaccharides possessed no biological violations, allowing them to meet the Lipinski rule and have good bioavailability scores (BAS = 0.55–0.56). This might be associated with their promising biological effects [39,40,41,42]. These results were confirmed by both bioavailability polygons and the model (Figure 4A,B). Furthermore, all the *S. tomentosum* monosaccharides were predicted not to permeate the blood–brain barrier (BBB) and have low gastrointestinal absorption, except rhamnose. To date, fifty-seven putative functional cytochrome P450 (CYP) isoforms have been enumerated, among which five principal isoforms (CYP1A2, CYP2C19, CYP2C9, CYP2D6, and CYP3A4) performed the metabolism of more than 80% of used drugs [43,44]. Hence, inhibiting these CYP isoforms may result in metabolic disruptions and toxic outcomes. Accordingly, it was found that all of the *S. tomentosum*-identified monosaccharides inhibited none of the studied CYPs. Thus, it could be deduced that *S. tomentosum* monosaccharides are not associated with disturbance of drug transport and metabolism [41,45,46]. Skin permeability of the studied monosaccharides, as assessed by log Kp, ranged between −8.3 and −9.7 cm/s, which indicates low skin permeation [40,47]. Nevertheless, the compounds were found to be easy to synthesize as the synthetic accessibility only ranged between 3.26 and 4.05.

### 2.4. Antioxidant Activity of PCT

**Figure 5:**
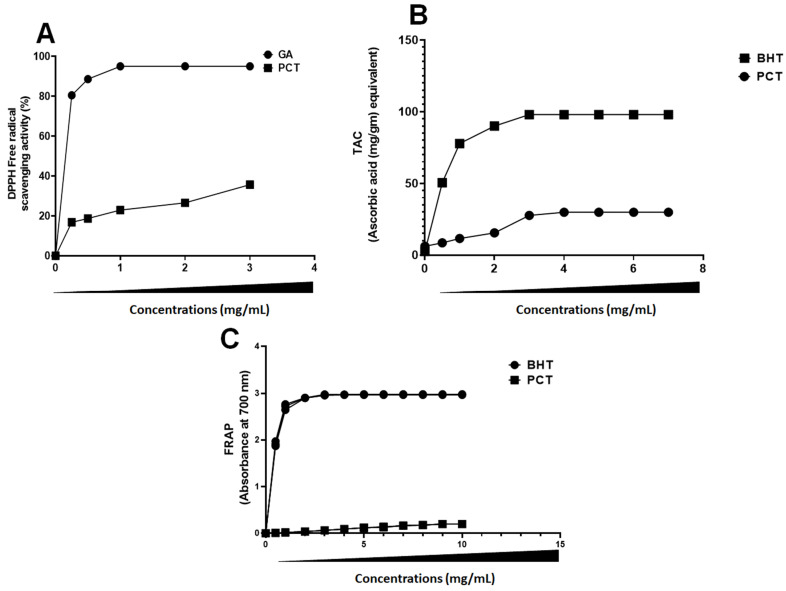
Antioxidant potentials of PCT. (**A**) DPPH radical scavenging activity. (**B**) Total antioxidant activity. (**C**) Reducing power. Gallic acid used as standard in DPPH test, and BHT used as standard in reducing power assay and total antioxidant activity.

The antioxidant activity of food extracts and nutritional additives significantly and immediately impacts cellular antioxidant activity. Their assessment offers one of the most effective ways to assess a biological molecular ability to prevent cellular milieu oxidation and subsequently prevent the emergence of health problems. In our study, to understand the possible antioxidant mechanism of PCT, several tests have been adopted to evaluate PCT’s antioxidant potential. PCT’s DPPH radical scavenging effect was studied and compared with the standard gallic acid. DPPH is a free radical compound. It has been used widely as a free radical to determine the scavenging ability of samples. Our results showed that the capacity of PCT to scavenge DPPH radicals was monitored in a dose-dependent manner. In fact, at the highest concentration, DPPH radical scavenging activity was 70.64% ± 0.75 (Figure 5A). However, gallic acid has a maximum percentage inhibition of 99.91 ± 0.09 at 0.5 mg/mL. This activity is low when compared with other extracts. For example, the IC50 DPPH of polysaccharide from *Caulerpa lentiffiera* was 74.03% ± 7.75 [48]. At a 2 mg/mL concentration, a polysaccharide from *Chaetomorpha linum* exhibited very high activity (62.98% ± 0.50), similar to gallic acid [29]. However, sulfated polysaccharides extracted from the green algae *Codium bernabei* have 36.13% scavenging activity of DPPH [36].

A significant reducing power activity can be identified when the solution’s yellow color changes to green due to the lowering of the Fe^3+^/ferricyanide complex to the ferrous form (Fe^2+^). As shown in Figure 5B, the thickening power of PCT saves a value of 0.2 (OD 700 nm) at a concentration of 10 mg/mL. In the same experimental condition, the reducing power of BHA, used as a standard positive control, reached about 1.5 (OD 700 nm). Following our results, polysaccharides extracted from *Ulva prolifera* at 150 °C and sulfur content 14.23–16.28% have about 0.2 OD at 700 nm [49]. The result of total antioxidant activity (TAC) is presented in Figure 5C. In our study, PCT reached the highest total antioxidant capacity (OD695 nm = 0.35) at a maximum concentration (7 mg/mL).

Interestingly, polysaccharides from green seaweeds exert direct antioxidant action based on the primary mechanisms of scavenging free radicals and inhibiting their appearance [12].

In this context, the antioxidant activity of polysaccharides is strongly related to several factors, such as molecular weight, monosaccharide composition, structure, conformation, polarity, and intramolecular hydrogen bonds [50]. The presence of rhamnose, galactose, arabinose, and mannose in polysaccharide fractions causes potent hydroxyl radical inhibition (Table 3). Moreover, as noted by Raposo et al. [51], there is a positive correlation between sulfate content and the scavenging power of polysaccharides. In our study, the powerful antioxidative properties of PCT are due in part to the number of monosaccharides (Figure 1B and Table 3) and their unique structural characteristics. Based on the SEM results, the ability of PCT to scavenge free radicals is probably related to its rough surface morphology. According to Ouahid et al. [52], *C. tomentosum* is classified as the second producer of sulfated polysaccharides. This sulfated polysaccharide can prevent the accumulation of free radicals and denature these reactive chemical species. The antioxidant activity of polysaccharides is probably related to their ability to be incorporated into cells and donate protons to free radicals to block their higher reactivity and convert them to more stable products unable to react with cellular macromolecules such as DNA, membrane lipids, and proteins [12].

The excellent ABTS^.^ activity, reducing power capacity, and chelating effect of polysaccharides have been discovered to be linked to their uronic acid, galacturonic acid, and glucuronic acid content. For example, the formation of a strong link between COOH/OH groups in uronic acid and Fe^2+^ was found to be responsible for the polysaccharide’s Fe^2+^-chelating ability [50]. In general, a more remarkable chelating ability is observed when a fraction of polysaccharide encloses a higher concentration of functional structure. Their presence was essential for successfully chelating unstable radicals [53]. Therefore, the synergic effects of different antioxidant mechanisms can be proposed as the main strategy of PCT for reducing the severity of cellular damage. As a result, the carbohydrate molecule extracted from *C. tomentosum* presents a powerful candidate that may serve as a defense mechanism against these causes of oxidative and radical damage and subsequently be used as an additive bioactive molecule in several food and pharmaceutical formulations.

**Table 3:** Relationship between sulfate contents and biological activities of marine algae polysaccharides.

Algae Species	Monosaccharide Unities	Sulfates Degree	Activities	References
*Chondrus canaliculatus*	arabinose mannose glucoronic acid	0.0025	antioxidant	[30]
*Chaetomorpha linum*	arabinose, mannose glucoronic acid	0.02	antioxidant	[29]
*Falkenbergia rufolanosa*	mannose glucuronic acid	5.97	antioxidant anti-inflammatory anti-coagulant	[54]
*Codium cylindricum*	mannose galactose arabinose glucose ribose	2.44	anti-coagulant anti-angiogenic	[27]
*Codium tomentosum*	arabinose fructose, xylose rhamnose glucoronic acid	5.30	anti-inflammatory antioxidant anti-angiogenic	our results

### 2.5. Effect of PCT on Inflammatory Edematous Symptoms

**Figure 6:**
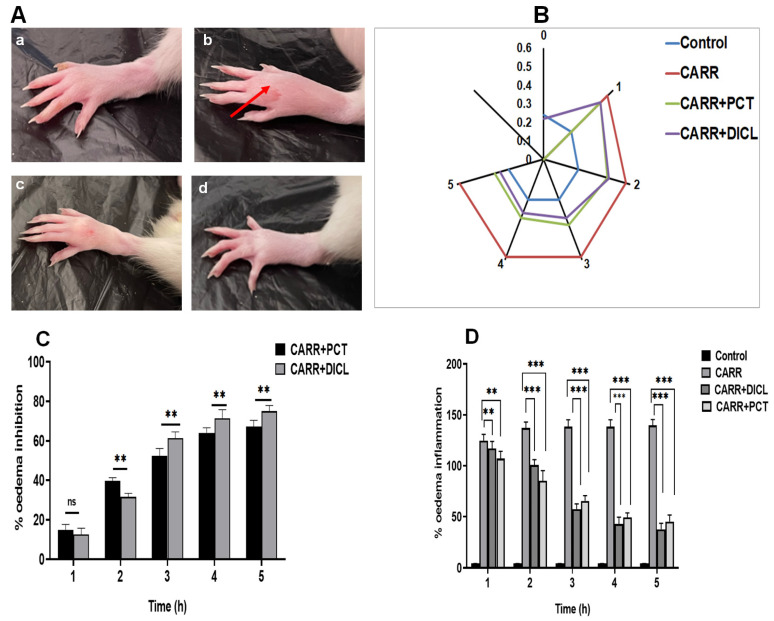
(**A**) Effect of PCT on paw edema in rats induced by carrageenan during 5 h: (**a**) control group, (**b**) CARR group, (**c**) CARR + PCT group, and (**d**) CARR + DICL group. Red arrows in the photos indicate edema on the paws of the rats. (**B**) Paw edema thickness (mm). (**C**) Percentage of edema inhibition. (**D**) Percentage of edema inflammation. Control: normal rats injected with NaCl 0.9%; CARR: rats injected with carrageenan 1%; CARR + PCT: inflamed rats treated with 20 mg/kg; CARR + DICL: inflamed rats treated with diclofenac 25 mg/kg. Values represent means ± SEM (n = 6) in each group; ns, no difference; ** *p* < 0.01; and *** *p* < 0.001.

The evolution of the size of swelling in control and treated groups for 5 h after injection of CARR is shown in Figure 6B. It reaches maximum size (0.58 ± 0.001 vs. 0.24 ± 0.005) after 3 h from the administration of CARR and was maintained until 5 h. Our results also showed that administration of PCT at the dose of 20 mg/kg significantly reduced the size of swelling in a time-dependent manner until the end of the fifth hour compared to the control group (*p* < 0.001). It gradually decreased to 0.44 ± 0.03, 0.39 ± 0.01, 0.35 ± 0.01, and 0.34 ± 0.01 at the end of 2, 3, 4, and 5 h, respectively (Figure 6B). In the same experimental condition, this reduction in the size of edema is very similar to that induced by DICL at the dose of 25 mg/kg.

As seen in Figure 6C,D, at the first hour of treatment, the percentage of edema inhibition and percentage of edema inflammation were 14.83% ± 2.70 and 107.15% ± 7.10, respectively. These two parameters progressed positively, registering values of 67.16% ± 3.20 and 45.16% ± 6.58 at the fifth hour (*p* < 0.001). The diclofenac-treated groups exhibited a significant decrease in paw edema (*p* < 0.001); the maximum percentage of edema inhibition was 75.01% ± 2.88, and edema inflammation was 37.58% ± 6.00. Treatment of several diseases strongly correlates with suppressing inflammatory symptoms; in this context, natural marine biomolecules present a new therapeutic option as anti-inflammatory agents. Our results correlated with various studies showing that polysaccharides derived from green seaweeds have great anti-inflammatory and antinociceptive activity. Polysaccharides from *Turbinaria decurrens* [34], Halimeda tuna [55], and *Caulerpa racemose* [56] were evidenced to offer the highest anti-inflammatory properties based on their capacity to reduce rat edema in an animal experiment.

Inflammation can be defined as a natural immune response against biological and chemical agents and physical and mechanical agents like tissue injuries, fractures, trauma, and ligatures [57]. The carrageenan-induced paw edema model usually evaluates the anti-inflammatory activity of drugs and new chemicals [58].

This model has been characterized as a biphasic event, with the first phase (1–2 h after carrageenan injection) and a late or second phase (2–6 h maximum action). The sequential release of inflammatory mediators marks these two phases. In the first phase of edema, histamine, serotonin, bradykinin, and substance P are released, increasing vascular permeability and blood flow. In the second phase, granulocyte cells are intentionally infiltrated into the site of inflammation. The chemical interaction between these cells is responsible for developing the inflammatory response [59].

Based on our findings, the maximum swelling size was observed after a 3 h CARR injection. That is why this time is considered a reliable time for showing the inflammatory activity of the test products, so the capacity of PCT to reduce the intensity of inflammatory reaction could be related to the inhibition of release and/or synthesis of inflammatory factors implicated in the second phase of the inflammatory response [15,22]. It is also important to note that several studies reported the capacity of polysaccharides to modulate the gene expression of cytokines, TNF-alpha, and other factors implicated in the inflammatory process [48,60,61]. Hence, the anti-edematous activity of PCT may be explained by blocking the production of these pro-inflammatory mediators and reducing their effects on hematological and biochemical parameters.

#### 2.5.1. Influence of PCT on Hematological Parameters

**Figure 7:**
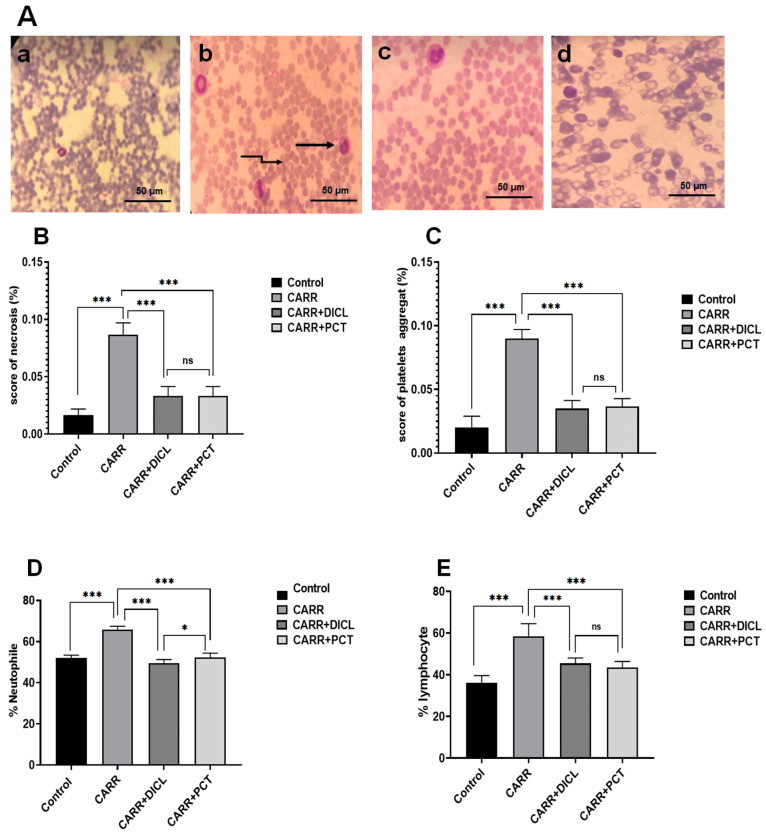
(**A**) Blood smears stained with May–Grünwald–Giemsa examined with a light microscope at ×400 magnification (scale bar = 50 μm): (**a**) control group, (**b**) CARR group, (**c**) CARR + PCT group, and (**d**) CARR + DICL group. The arrows indicate 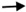 lymphocytes and 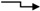 platelet aggregates. (**B**,**C**) Semi-quantitative scores of necrosis and platelet aggregates in the blood smears of adult rats, respectively. (**D**,**E**) Effect of PCT on lymphocytes and neutrophil cell infiltration in carrageenan-induced paw edema, respectively. Values are expressed as means ± SD; ns, no difference; * *p* < 0.05 and *** *p* < 0.001.

The induction of acute inflammation in the paws of rats disrupted hematological parameters. Hence, the WBC, RBC, and PLT counts of all the groups were analyzed. As shown in Table 4, after 5 h from the administration of CARR, an appreciable increase in WBC (*p* ≤ 0.001) and PLT counts was observed compared with the control group. These results are also shown in a blood smear by the abundant presence of WBC and PLT (Figure 7A,D,E). The quantitative analysis of blood smears is based on the above results (Figure 7B,C). Under the same experimental conditions, the RBC count in rats with inflammation was lower than that in normal rats. Treating rats with PCT and DICL improved the WBC, RBC, and PLT counts (*p* ≤ 0.01) compared to the CARR group. In addition, no significant difference was shown between the PCT and DICL groups. It is interesting to clarify that all drugs do not affect HCT, Hb, MCV, and MCH levels (*p* > 0.001).

Hematological parameters are among the most sensitive inflammatory marker responses [62]. The peripheral blood counts and morphology of cells may be utilized to determine the severity of the inflammation. In the present study, the decrease in the RBC levels is correlated with the oxidative stress situation. In fact, during inflammation, the excessive generation of ROS influences the deformability of RBCs and enhances their degradation. According to some theories, a significant factor causing cell and tissue damage, including cancer and inflammatory illnesses, is the excessive generation of ROS at the site of inflammation [40,63].

On the other hand, the increase in the WBC and platelet aggregation refers to many complex mechanisms implicated in the inflammatory process. Macrophages, basophils, mast cells, neutrophils, and eosinophils present the first line of defense against pathogens and act as reservoirs for soluble mediators [64]. The release of a vasoactive compound like histamine, serotonin, prostaglandin, and leukotriene by macrophages in response to physical stress seen in the tissues at the outset of an infection (heat, cold, pressure) increases vascular permeability and vasodilatation [65,66]. Platelets and granulocytic cells are activated and then migrate from the blood to the injury site. Platelets, anucleate circulating cell fragments implicated with other proteins such as fibrinogen and vitronectin in the coagulation system, also have inflammatory functions. They are quickly activated to produce numerous mediators, such as heparin and serotonin, which significantly affect the vasodilatory status of the acute vascular response [64,67]. There is a strong relation between the reduction in edema volume and the level of WBCs in blood. The powerful effect of PCT in reducing edema size after 2 h of the inflammatory reaction’s stalling is proportional to the regeneration of WBC count. PCT can directly affect the production of soluble mediators that stimulate and regulate the inflammatory response and activate inflammatory cells [12,61].

**Figure 8:**
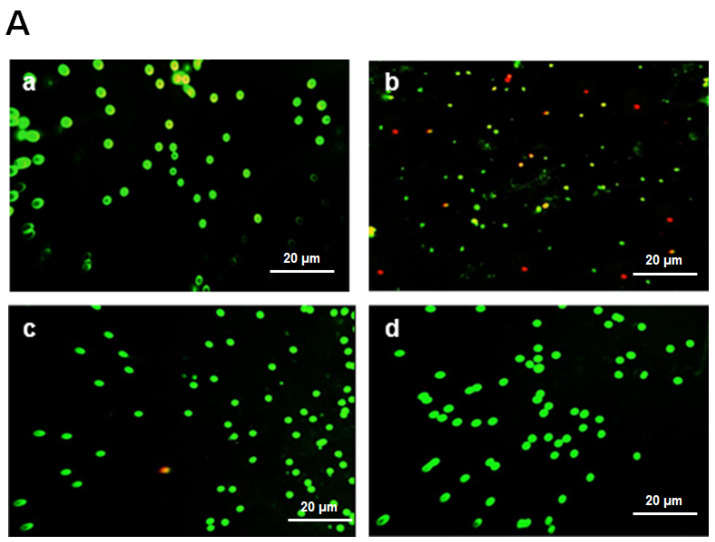

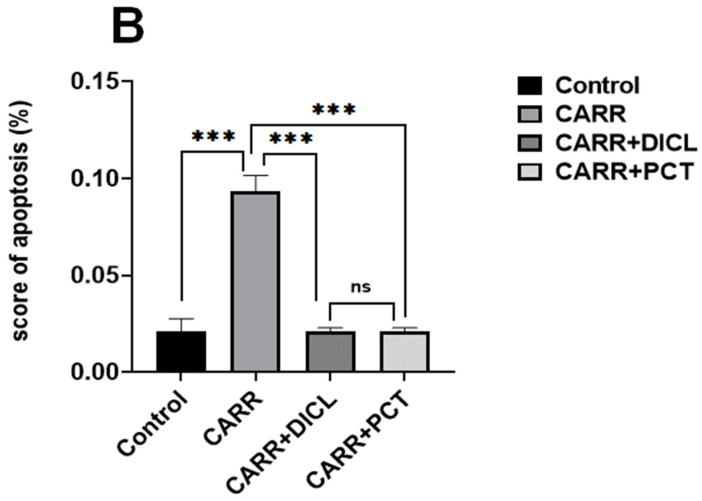
(**A**) Effect of inflammation induced by CARR and treatment with PCT or DICL on genetic materials of white blood cells after 5 h based on MN test assay (scale bar = 20 μm): (**a**) control group, (**b**) CARR group, (**c**) CARR + PCT group, and (**d**) CARR + DICL group. Green spot indicates intact DNA, and the red spots reflect damaged DNA. (**B**) Semi-quantitative scores of apoptosis of adult rats. Values are expressed as means ± SD. ns, no difference *** *p* < 0.001.

The morphology of the white cell nuclei of all groups (control, CARR, CARR + PCT, and CARR + DICL) is presented in Figure 8. Compared to the control group, treatment of rats with CARR causes a total fragmented nuclear morphology, as shown by red/orange fluorescence (damaged DNA). The administration of PCT at the dose of 20 mg/kg reduces the frequency of DNA damage significantly, as shown by the increase in green/yellow fluorescence (intact DNA) (Figure 8A,B). Free radicals can target all constituent cells and induce lipid/protein peroxidation and DNA oxidation. Therefore, the formation of a DNA smear may be considered a hallmark feature of necrosis and chromatin degradation in WBCs and inflamed tissue cells [68]. Several studies have noted that the in vitro antioxidant activities of polysaccharides from algae are related to their capacity to protect cells against oxidative stress. In this context, polysaccharides of *Typhaan gustifolia* play a significant role as a protecting agent against ROS generation in RAW264.7 cells [19].

#### 2.5.2. Effect of Inflammation on Serum Protein Levels

The body’s initial response to antigenic stress is mediated by acute-phase proteins (APPs), which also serve as the basis for the innate response, and most of them are produced in the liver, particularly from the supply of amino acids created by the reduction in albumin production [57]. APPs are blood proteins produced during the acute-phase response, can interact with both pathogen elements and cells to modulate the inflammatory response, and are susceptible to identifying a wide range of disorders that affect an animal’s health and recognize inflammation or infections. As di Filippo et al. [69] reported, APPs are immediately and accurately used to show the presence of inflammatory and infectious diseases. In the present study, albumin alpha, beta, and gamma globulins were identified by routine electrophoresis. As shown in Table 5, injection of CARR in the paw of rats significantly increased the level of total protein in the blood (*p* < 0.001) when compared with the control group (64 ± 0.1 g/L vs. 56.46 ± 0.64). However, we noted a significant decrease in the albumin level (*p* < 0.001). Albumin is the most abundant protein in human blood as the primary reservoir of amino acids, and it is implicated in several functions such as osmotic pressure regulation, maintenance of blood pH, transport of many hydrophobic ions and molecules (minerals, hormones, vitamins, and fatty acids) [64]. This protein fraction is defined as a negative marker for inflammation. Based on our observations, the demand for amino acids is significantly raised during the acute-phase response. Generally, 30–40% of hepatic protein anabolic capacity is used for the synthesis of the positive acute-phase proteins [70]. Thus, albumin is downregulated as a significant source of amino acid production.

The liver generally secretes alpha, beta, and gamma globulins, which are frequently used as a good index for many infectious diseases. The gamma fraction is one of the most sensitive markers of inflammation, and it is widely used to determine the severity and type of the immune reaction. Most immunoglobulins are part of the gamma fraction, but IgM, IgA, and some IgGs are defined as beta globulins. This type of globulin also complements CRP, fibrinogen, and ferritin. Similarly, alpha globulins represent a heterogeneous group of proteins localized in the endoplasmic reticulum of hepatocytes. A perturbation of the levels of globulins may be used to determine an inflammatory reaction [57,64]. As depicted in Table 5, the concentration of alpha 1, alpha 2, beta 1, beta 2, and gamma globulins showed a significant increase in the CARR group compared to the control. Alpha2 and beta1 globulin presented the highest values (12.55 ± 0.25 and 7.30 ± 0.20), followed by gamma globulin (7.41 ± 0.08).

Furthermore, treatment with PCT and DICL has significantly reduced CAR-induced elevation in these parameters. This finding can be related to many causes and is consistent with the adverse effects of CARR on the immune system and liver cells. Acute-phase proteins are crucial to the progression of the inflammatory response at different phases. According to previous studies, all perturbations noted in the protein profile could be explained by the effects of chemical mediators of the inflammatory reaction [57]. Cytokine signals from the inflammation site directly influenced APP production and release. The stimulation of immune cells, especially macrophages, induced an excessive production of pro-inflammatory mediators. This molecule acts on the liver and disrupts the synthesis of APPs, which is implicated in the regulation of immune response; however, with prolonged stimulation, these proteins alter homeostasis and can lead to chronic inflammation, tissue damage, and metabolic disturbances [64].

Interestingly, PCT reduced these inflammatory indicators, which had risen after the inflammatory response. The alleviative effect is certainly related not only to PCT’s capacity to regulate the synthesis of APPs in the liver but also to the decrease in the stimulation of immune cells and the production of pro-inflammatory mediators. Thus, it indicates that tested polysaccharides have potential effects as regulators of acute inflammation.

#### 2.5.3. Evaluation of Oxidative Stress Parameters in Erythrocytes and Edema Tissue

Several natural treatments are known to downregulate inflammation. However, the involvement of the antioxidant mechanism route in mediating the effect of such anti-inflammatory medications has yet to be clarified. Hence, to control inflammation, it is necessary to study the cellular state of the pro-/antioxidant system. Indeed, an imbalance in the redox state can induce severe cellular complications until cell death. The antioxidant result is displayed in Table 6.

Lipid peroxidation (LPO) and protein oxidation (AOPP) were used to control cell function under oxidative stress conditions. Malondialdehyde (MDA) and AOPP levels were evaluated in the tissue homogenate of rat paws and erythrocytes. After 5 h of injection of CARR, the levels of MDA and AOPP showed a significant increase (*p* < 0.001) compared with the control group (Table 6). However, in animals treated with PCT and DICLO, the MDA and AOPP show a remarkable 25–36% and 33–90% decrease, respectively (*p* < 0.001). These results indicate that PCT can protect skin cells against inflammation-induced lipid and protein peroxidation. Holanda et al. [71] explored the impact of zymosan-induced inflammation on oxidative stress parameters. They illustrated that excessive ROS production at the site of inflammation enhances the lipid peroxidation process. Moreover, Mzid et al. [65] clarified the strategy for CARR-induced inflammation, which principally involves oxidative damage to cellular proteins and lipids. Wei et al. [19] also investigated the effects of inflammation on ROS levels. They illustrated that LPS treatment increased ROS content in RAW264.7 cells.

The induction of a rapid, non-specific inflammatory reaction in a rat leg after CARR injection may increase the consumption of the immune cells in oxygenate [57]. Indeed, macrophages require oxygen to produce inflammatory mediators such as cytokines and interleukins, and subsequently, ROS production is responsible for the apparition of oxidative stress [39]. The major ROS generated are superoxide anions, hydroxyl radicals, nitric oxide, and peroxides, and they can induce extensive damage to cell structures and tissues [60]. Lipids located at the cell membrane level have preferential targets for ROS since they are rich in PUFAs. Lipoperoxidation could have peroxides and aldehydes like MDA as products [72].

Superoxide dismutase (SOD) is a metalloprotein that converts superoxide anion to oxygen (O_2_) and hydrogen peroxide. Compared with the control group, CARR injection significantly reduced SOD levels in paw edema and erythrocytes (*p* < 0.001). However, treating animals with PCT increased the SOD activity in paw edema and erythrocytes by 24% and 47%, respectively. PCT- and DIC-treated groups showed the same SOD activity in all the samples.

Glutathione peroxidase (GPx) plays an important role in the reduction of hydroperoxides and in preventing the oxidation of lipids and proteins. GPx activity is significant in controlling oxidative stress in the cell. In our study, the GPx level was examined in the tissue homogenate of rats. CARR injection (group 2, *p* < 0.001) resulted in a significant decrease in activities of GPx by 57% and 42% when compared with the control group. On the other hand, co-administration of PCT ameliorated the effects of inflammation and significantly increased (*p* < 0.001) GPx activity compared to the CARR group.

However, treatment with CARR elevated GSH levels compared to the control group (*p* < 0.001). Co-treatment with PCT and CARR resulted in a significant decrease in GSH concentration compared to the CARR group (*p* < 0.001).

During the inflammatory process, enzymatic (SOD, CAT, GSH-Px) and non-enzymatic antioxidant defense systems, such as GSH, play a significant role as scavengers of free radicals to protect cells against oxidation [34]. However, excessive production of ROS weakens antioxidant enzymes, distorts proteins, and induces global cell dysfunction, followed by a state of apoptosis through the oxidation of DNA, protein, and lipids [73]. The present study’s findings are consistent with the previous investigation of Bhardwaj et al. [62], which showed that inducing inflammation by injection of CARR reduced the activity of GSH and Myeloperoxidase (MPO). Manikandan et al. [57] showed that the development of an inflammatory reaction in rat skin induces a remarkable decrease in the enzymatic activity of GPx, catalase, and SOD. On the other hand, co-treatment with PCT stabilizes the activities of cellular oxidative stress and has been considered as the therapeutic strategy of PCT against inflammation [61].

These observations are in line with those found in the study of Jaballi et al. [30] and Feki et al. [54], which revealed that polysaccharides from *Chondrus canaliculatus* and *Falkenbergia rufolanosa* have a great capacity to eliminate oxidative damages. Thus, PCT performs its anti-inflammatory activity directly as a complex of antioxidant molecules and indirectly by stimulating antioxidant enzymes.

These data are in agreement with the literature. Polysaccharides from macro- and microalgae show an increased ability to prevent the oxidation of cellular constituents by inhibiting the production of superoxide anion radicals, hydroxyl radicals, and lipid peroxidation based on their antioxidant activities [24,61].

#### 2.5.4. PCT Attenuates Histopathological Alterations

**Figure 9:**
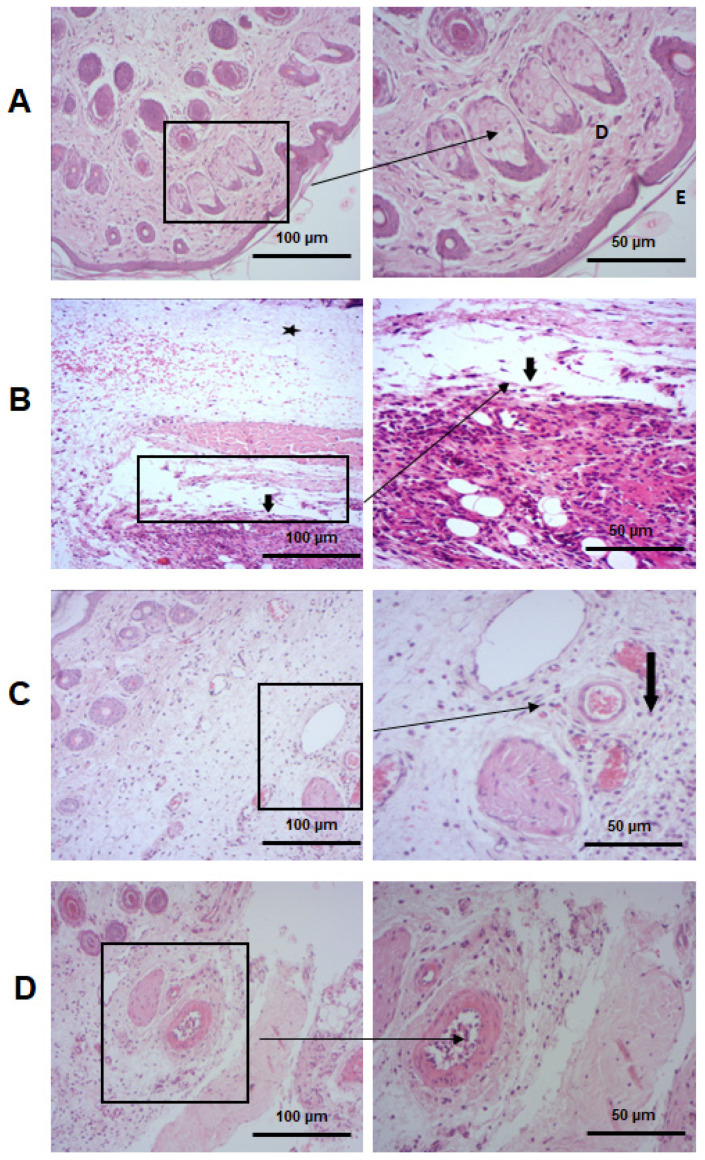

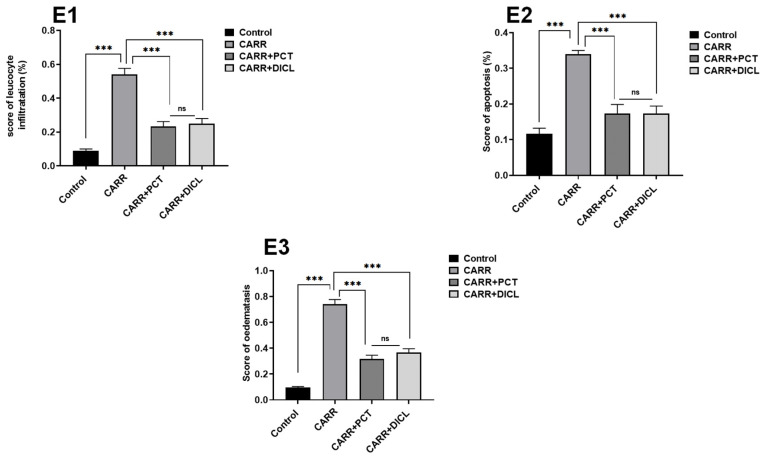
Effect of PCT and DICL on CARR-induced histological modification in rat skin. H&E-stained skin sections were examined with a light microscope at ×50 (scale bar = 100 μm) and ×200 magnifications (scale bar = 50 μm). (**A**) Tissue sections of skin rats from the control group; (**B**) tissue sections of rats from the CARR group; (**C**) tissue sections of skin rats from the PCT group; and (**D**) tissue sections of rats from the diclofenac group. Arrows indicate the following: D: dermis, E: epidermis, 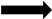 lymphocyte infiltration, 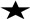 edema. (**E1**–**E3**) Score of inflammatory infiltrate, apoptosis, and edema. Significance: ns: no difference; *** *p* < 0.001.

The biochemical disturbances observed in our study are correlated with histological data. The control group shows a normal skin histoarchitecture with some inflammatory cells identified as tissue-resident cells (Figure 9A). Four hours after the induction of inflammation, a microscopic study of paw tissue showed the presence of acute edematous in the epidermis and dermis with a spongy appearance. Vasculitis and hyperemia were observed around the vessels, which indicates increased blood flow to inflamed tissue. Histological analysis also showed a significant increase in the migration of leukocytes to the sites of inflammation (Figure 9B). The diapedesis phenomenon explains the observed leukocyte load. Inflammatory chemical signals are released during inflammatory reactions to recruit blood immune cells to damaged tissues [64]. Co-administration of PCT at 25 mg/kg alleviates tissue damage (Figure 9C) with a notable reduction in leukocyte migration, edematous, and hyperemia. Souza et al. [74] explained that polysaccharides extracted from *Morinda citrifolia* Linn decreased the migration of leukocytes to the peritoneal cavity of the mice after CARR injection. According to the histopathological score in Figure 9E, the group treated with PCT displayed the lowest score for leukocyte infiltration, edematous, and necrosis. These data indicated the powerful capacity of PCT to reduce the severity of inflammation. The anti-inflammatory effect of PCT is mainly based on macrophage modulation.

Macrophages and polymorphonuclear cells have a central role and are the main immune cells that recur during an inflammatory reaction. They are responsible for the secretion and regulation of the chemical mediators involved in the evolution of the immune response. These cells are one of the initial barriers against infections [51,61]. Based on this information, PCT’s anti-inflammatory activity may be explained by its ability to interfere with the migration of leukocytes to the site of inflammation, minimize the production of pro-inflammatory cytokines, and subsequently decrease pain, oxidative stress, and tissue damage [19]. Albuquerque et al. [20] explained that the heterofucan from *D. menstrualis* can bind directly to the cell surface of leukocytes, especially polymorphonuclear cells (PMNs), and decreases inflammation. Therefore, polysaccharides promote a positive response in inflammatory reactions by inhibiting chemo-cellular cooperation.

### 2.6. Ex Vivo Anti-Angiogenic Effect of PCT

A substance’s capacity to have a biological effect is influenced by its efficacy and potency. This effect is usually determined using a dose–response relationship on a biological target. In the past decade, numerous studies have shown the interdependence of angiogenesis and inflammation in the development of several diseases. Therefore, efforts have concentrated on creating new, powerful anti-inflammatory and anti-angiogenic medications. The CAM is an original in vivo model for assessing neovascularization using fertilized chicken eggs. It exhibits a biological substance’s capacity to promote or inhibit the growth of blood vessels and identifies its pro- or anti-angiogenic properties.

**Figure 10:**
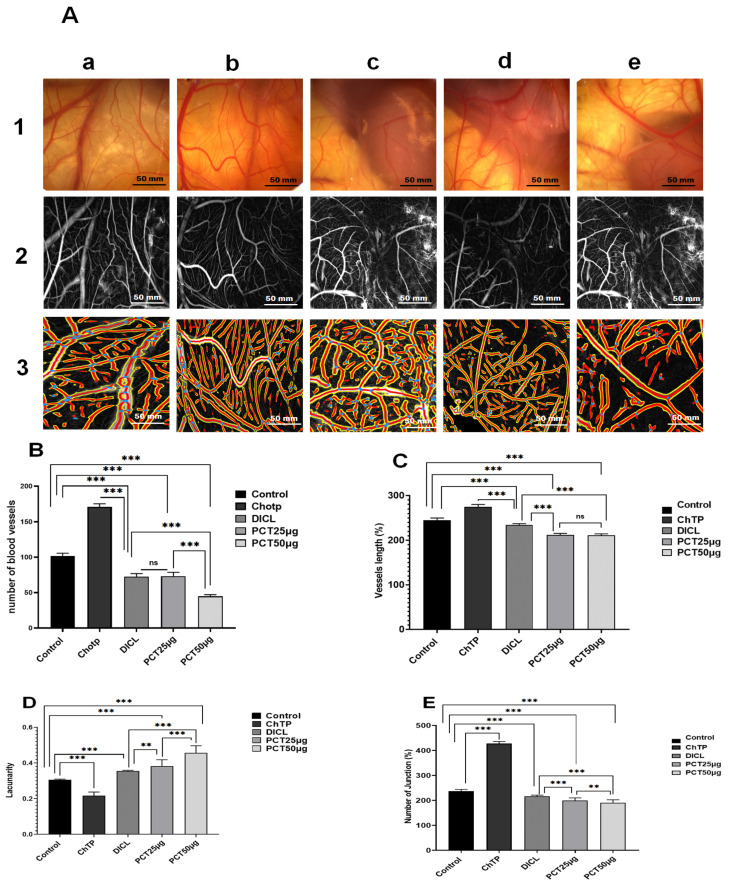
Effect of PCT on CAM angiogenesis (**A**) Morphological micrographs: (a) negative controls; (b) positive control (CAM treated with choriogonadotropin (0.5 μg/g eggs)); (c) negative control (CAM treated with diclofenac (5 μg/g eggs)); (d,e) CAM treated with 25 μg and 50 μg/g eggs of PCT (scale bar = 50 mm); 1: photographs of blood vessels; 2: photomicrographs treated with ImageJ software 3: photomicrographs treated with angiotools software. (**B**) Number of vessels. (**C**) Length of blood vessels. (**D**) Lacunarity. (**E**) Number of junctions. Values are expressed as means ± SD for 6 eggs in each group. ns: no difference; ** *p* < 0.01, and *** *p* < 0.001.

As shown in Figure 10A, blood vessels formed dense branching vascular networks in the negative control. CAM neovascularization, defined as the formation of new vessel numbers and branching patterns, was decreased by PCT in a dose-dependent manner. Compared with the control group, at the doses of 25 μg and 50 μg, the number of vessels revealed a significant reduction (*p* < 0.05) (Figure 10B). Moreover, the mean length of the blood branches of groups treated with 25 and 50 μg declined reasonably (*p* < 0.001) when compared to the control group (Figure 10C). In addition, a decrease in the number of junctions was observed for the groups treated with 25 μg and 50 μg of PCT in comparison to the control group (Figure 10E). As expected, a higher value of lacunarity was found (Figure 10D) for PCT 25 μg (0.38 ± 0.03) and PCT 50 μg (0.42 ± 0.04). These results proved that PCT significantly inhibited the angiogenesis of CAM with a score value of 2 (Table 7).

The formation of new blood vessels from preexisting vascular presents one of the most dynamic proliferation and differentiation processes [4]. It implicates the degradation of extracellular matrix (ECM), endothelial cell proliferation, migration, differentiation, tube formation, and sprouting of new capillary branches. Recently, numerous studies proved that algae such as *Grateloupia longifolid*, *Sargassum stenophyllum*, *Fucus vesiculosus*, *Codium cylindricum*, and *Laminaria japonica* are a significant source of anti-angiogenic compounds [23,75]. Meanwhile, several polysaccharides have anti-angiogenic properties. For example, the sulfated derivative of a heteropolysaccharide from *Chrysanthemum morifolium* has been reported to have anti-angiogenic activity in an HMEC-1 cell model [76]. Sulfated galactan isolated from *Codium cylindricum* has a higher capacity to suppress micro-vessel formation in an ex vivo serum-free matrix culture model using a rat aortic ring [12,23]. Structural characteristics of polymers, including sugar composition, linkages, and molecular structures, are correlated with anti-angiogenesis activities (Table 2). In our experiments, a polysaccharide from *C. tomentosum* markedly suppressed the formation of new vascular networks in a dose-dependent manner as assessed by an ex vivo model. This finding supports the idea that PCT has powerful anti-angiogenic effects, and clinical trials can be conducted to examine their mechanism of action and their capacity to interfere with factors that regulate the angiogenic process. Multiple cell factors are implicated in the angiogenesis signaling pathway [5]. The VEGF and bFGF family of proteins are glycoproteins responsible for increasing levels of pro-angiogenic signal proliferation [77]. The suppression signal of these factors can significantly reduce the neovascularization process. In this context, PCT presents a compound that directly blocks ex vivo angiogenesis. The anti-angiogenic effect of PCT, which is made up of units of glucose, galactose, and fructose, can be related to their capacity to inhibit angiogenic growth factors. According to Liu et al. [78], fucoidan from *Undaria pinnatifida* reduced micro-vessel outgrowth via suppression of VEGF-A expression. This finding confirmed the strategy of using polysaccharides to prevent tumor migration and metastasis with minimum side effects and higher efficacy.

On the other hand, there is a strong relationship between inflammation and the angiogenic process. In this context, Xiong et al. [79] demonstrated that polysaccharides from the flesh of *Cipangopaludina chinensis*, used as anti-inflammatory and anti-angiogenic agents, can reduce the release of some inflammatory factors and consequently reduce inflammation. Sulfated polysaccharides obtained from *Amansia multifida* algae significantly decreased angiogenesis in chicken chorioallantoic membranes [16].

## 3. Materials and Methods

### 3.1. Algae Collection and Species Identification

**Figure 11:**
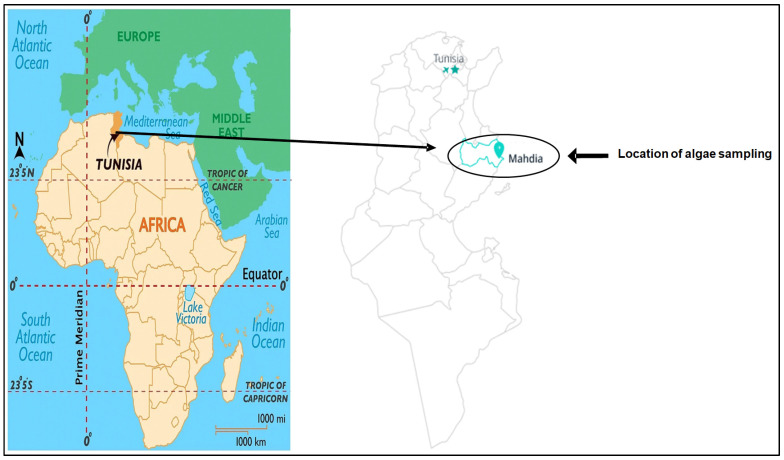
Location of the algae collection site (Chebba, Mahdia, Tunisia).

The algae were collected from Chebba, Mahdia, Tunisia (Figure 11), in March 2022. The species was identified at the National Institute of Marine Sciences, Sfax-Tunisia.

### 3.2. Polysaccharide Extraction

After collection, the seaweed samples were transported to our laboratory and immediately washed twice with distilled water to remove grains of sand, epiphytes, and other impurities. Subsequently, the alga was dried from the open area and out of the light for two weeks. It was crushed to obtain a fine powder and then stored in glass bottles at room temperature. The procedure for extracting polysaccharides was performed according to the method developed by Liu et al. [80] with slight modifications. In the first step, the powder of *C. tomentosum* (70 g) was washed with 1 L of absolute ethanol to remove pigmentation. After that, the depigmented samples were treated twice with di-ionized water at 90 °C for 4 h under agitation. The extracts obtained were filtered several times and then evaporated under a vacuum. The polysaccharide in the extracts was precipitated using ethanol 95% (1 V/3 V) at 4 °C for 24 h. Then, the solution was centrifuged at 8000 rpm for 15 min using a refrigerated centrifuge (Hettich Zentrifugen, ROTINA 380R, Berline, Germany). After the resulting extract was kept at 4 °C overnight, the final residue was re-dissolved in double-distilled water, and finally, the polysaccharides were lyophilized and stored at −20 °C. The extraction yield is expressed according to the following formula:

Extraction yield (% *w*/*w* = dry weight of the polysaccharide extracted (g)/dry weight of *C. tomentosum* (g)

### 3.3. Analysis of Biochemical Composition

The phenol sulfuric acid method was used to determine the total sugar content [81]. The barium chloride gelatin method was used to estimate the free sulfate present in polysaccharides [82]. Soluble protein contents were determined by a colorimetric assay using bovine serum albumin (BSA) as the standard [83].

### 3.4. Polysaccharide Spectroscopic Analysis

#### 3.4.1. UV Absorption Peak Detection and X-ray Diffraction

A final polysaccharide solution of 1% (*w*/*v*) was prepared using distilled water [84]. The UV absorption spectrum of the samples was recorded at 25 °C in the wavelength range of 200–800 nm using a UV–vis spectrophotometer (JENWAY/7315, Birmingham, UK).

The crystalline structures of PCT were evaluated at room temperature using an X-ray diffractometer (D8 advance, Bruker, Berline, Germany). The data obtained were collected in 5–80° with a step size of 0.05° and a counting time of 5 s/step.

#### 3.4.2. Scanning Electron Microscopy (SEM)

The microstructure of PCT was identified by SEM (Termosientific 250 microscope, Hitachi, Tokyo, Japan) operating at 3.0 Kv. The fraction analyzed was photographed with an angle of 90° to the surface.

#### 3.4.3. Monosaccharide Analysis by HPLC-FID

The HPLC-FID assay was performed as described by Bayar et al. [85]. The polysaccharide (2 mg) was hydrolyzed in 4 mol/L TFA at 100 °C for 8 h. Then, 20 μL of hydrolysate was added to 980 μL of H_2_O and filtered through a 0.45 mm pore size filter. Monosaccharide composition was analyzed by HPLC using an Aminex HPX-87H column (Bio-Rad Laboratories, Hercules, CA, USA) with H_2_SO_4_ as a mobile phase of (0.001 N), a flow rate of 06 mL/min, and a column temperature of 60 °C.

### 3.5. Druggability, Bioavailability, and Pharmacokinetic Properties

Both druggability and pharmacokinetic properties of the PCT-identified monosaccharides were assessed based on the ADMET properties (absorption, distribution, metabolism, excretion, and toxicity) as previously reported [39,40,47]. The bioavailability of the PCT-identified monosaccharides was also assessed based on the physicochemical structures [39,40,42].

### 3.6. In Vitro Biological Activity

#### 3.6.1. DPPH Free Radical Scavenging Assay

The DPPH radical scavenging activity of PCT was evaluated by 1-1-Diphenyl-2-picryl-hydrazyl (DPPH) following the method described by Lopes-Lutz et al. [86]. Different concentrations of PCT (0.25–10 mg/mL) were incubated with DPPH• solution (0.2 mmol/L of ethanol) for 30 min in the dark. The absorbance was determined at 517 nm with a spectrometer. The percentage of inhibition (PI) was calculated using the following equation:

PI (%) = (Ac − As) /Ac × 100

Ac: the absorbance of the control; As: the absorbance of the sample. Gallic acid was used as standard, and all experiments were performed in triplicate.

#### 3.6.2. Determination of Total Antioxidant Capacity

Total antioxidant capacity was determined according to Prieto et al. [87]. The assay is based on the capacity of the sample to reduce Mo (VI) to Mo (V) and the subsequent formation of a green phosphate/Mo (V) complex at acid pH. The intensity of coloration is directly proportional to the antioxidant capacity of the sample test. Briefly, 1 ml of reagent solution (0.6 M sulfuric acid, 28 mM sodium phosphate, and 4 mM ammonium molybdate) was added to 500 μL of samples and incubated at 95 °C for 90 min under a water bath; after cooling to room temperature, the optical density of the sample was measured at 695 nm.

#### 3.6.3. Determination of Reducing Power

The reducing power of the PCT was evaluated by the following method of Yıldırım et al. [88]. Samples of different concentrations were incubated with potassium ferricyanide (1% *w*/*v*) at 50° C for 20 min. After incubation, 0.5 mL of 10% trichloroacetic acid (TCA) was added, and the reaction mixtures were then centrifuged for 10 min at 3000 rpm. From each sample, 1.25 mL of the supernatant solution was mixed with 1.25 mL of distilled water, and then 0.25 mL of 0.1% ferric chloride was added. The absorbance of samples was calculated at 700 nm. Butylhydroxytoluene (BHT) was used as a standard antioxidant compound.

#### 3.6.4. Energy Value

The energy value of PCT was determined in kcal/g by multiplying the percentages of protein and carbohydrate with the recommended factors (2.44 and 3.57, respectively) [89].

### 3.7. Anti-Inflammatory In Vivo Assay

#### 3.7.1. Animals

The experimental procedures were carried out according to the Natural Health Institute of Health Guidelines for Animal Care and approved by the local Ethical Committee (Protocol No. 06.0003/23). All animal procedures were conducted strictly according to the “Institute’s ethical committee guidelines” for the care and use of laboratory animals. In the present study, 24 adult albino Wistar female rats with an initial weight of 150 ± 20 g were used. Female rats were obtained from the Institute of Pasteur, Tunisia. They were housed in plastic cages and fed with standard commercial pelleted feed, and water was supplied ad libitum. A constant light/dark cycle at a temperature of 22 ± 2 °C and a relative humidity of 45% was applied.

#### 3.7.2. Carrageenan-Induced Paw Edema

The female rats were divided into four groups of six individuals. The experimental groups were designed as follows:

The 1st group (control) was used as a negative control, receiving only physiological saline (0.9%) as a vehicle by gavage.

The 2nd group (CARR) was injected only with carrageenan (0.1 mL of 1% *w*/*v*) in the paw.

The 3rd group (CARR + DICL) was given 0.1 mL of diclofenac (25 mg/kg) by oral gavage.

The 4th group (CARR + PCT) was given 0.1 mL PCT (20 mg/kg) by oral gavage.

One hour after the respective drugs were administered, paw edema in the right hind paw of each animal was induced by subcutaneous administration (s.c.; intraplantar) of 0.1 mL of 1% freshly prepared solution of CARR in saline solution.

Paw edema thickness was measured at T = 0 min using a digital caliper before the carrageenan injection and considered as the initial volume (V0). After CARR injection, V_i_ was measured at 1, 2, 3, 4, and 5 h.

The percentage of edema inhibition/inflammation was determined according to the following formula:

Percentage of edema inhibition = [(V_0_ − V_i_)_control_ − (V_0_ − V_i_)_treated_]/(V_0_ − V_i_)_control_ × 100

Percentage of inflammation = [(VT − V0)/V0 × 100]

#### 3.7.3. Blood and Tissue Sample Collection

At the end of the experiment, the 24 rats were anesthetized with 10% chloral (0.3 mL per 300 g body weight) and sacrificed. Some blood samples were immediately collected in EDTA tubes for the determination of hematological parameters and cell viability and for the MN test. Serum samples were used to determine the acute-phase protein (APP) levels. After the dissection of the animals, paw tissue was stored in the freezer at −80 °C to determine oxidative stress parameters. The remaining tissue fragments were fixed in 10% formalin for histological examination.

#### 3.7.4. Determination of Hematological Parameters

Red blood cells (RBCs), white blood cells (WBCs), platelets, hemoglobin, hematocrit, mean corpuscular volume, and mean corpuscular hemoglobin were assayed by an electronic automated Coulter MAXM (Beckman Coulter, Inc., Fullerton, CA, USA). Blood smear slides were prepared, colored with May–Grunwald and Giemsa solutions, and visualized under an optical microscope at 400× magnification.

#### 3.7.5. Exploration of Oxidative Stress

Evaluation of lipid peroxidation: Malondialdehyde (MDA) is one of the end products formed during the decomposition of polyunsaturated fatty acids (PUFAs) mediated by free radicals. It is the most widely used lipid peroxidation marker, mainly because of the simplicity and sensitivity of the assay method. Briefly, 0.5 mL of tissue homogenate was mixed with 1 mL of 5% trichloroacetic acid and centrifuged at 2500 g for 10 min, and then 500 μL of supernatant was mixed with 1 mL thiobarbituric acid reagent (0.67%) and incubated at 90 °C for 15 min. The absorbance of the mixture was read at 532 nm in a UV spectrophotometer, lipid peroxide levels were expressed in nmol of MDA/mg of protein, and 1’, 1’,3’,3’-tetra methoxy propane was used as a standard [90].

Advanced oxidation protein product (AOPP) level: AOPP levels were analyzed according to the method of Witko et al. [91]. The tissue homogenate (0.4 mL) was mixed with 0.8 mL of TP (0.1 M; pH 7.4). Then, 0.1 mL of potassium iodide 1.16 M (KI) and 0.2 mL of acetic acid were added after 2 min. The absorbance of the reaction mixture was assessed at 340 nm. Each AOPP sample’s concentration was determined using the extinction (261 cm^−1^mM^−1^), and the findings were represented in μmoles/mg of protein.

Superoxide dismutase (SOD) enzyme activity: SOD enzyme activity was determined by a colorimetric method according to Beauchamp and Fridovich [92]. Briefly, 50 mM of tissue homogenates was added to potassium phosphate buffer (pH 7.8), 0.1 mM EDTA, 13 mM L-methionine, 2 μM riboflavin, and 75 mM nitroblue tetrazolium (NBT). The activity of SOD was measured at 560 nm and expressed as U/mg of protein.

Measurement of glutathione peroxidase activity: Glutathione peroxidase (GPx) activity in tissue was measured according to Flohé and Günzler [93]. One milliliter of the reaction mixture (2 mM glutathione (GSH), sodium azide (10 mM), and H_2_O_2_ (1 mM)) was added to 0.3 mL of tissue homogenates. TCA 5% was used to block the reaction. After centrifugation at 1500 g for 10 min, 0.7 mL of DTNB (0.4 mg/mL) was added to 0.2 mL of supernatant. Absorbance was measured at 420 nm, and the enzyme activity was expressed as nmoles of GSH/min/mg of protein.

Determination of GSH concentration: The GSH level in tissue homogenates was determined according to the method of Moron et al. [94]. The thiol function of GSH can react with DTNB, which induces the formation of a yellow-colored complex and TNB. Briefly, 500 μL of tissue homogenate in phosphate buffer was added to 3 mL of sulfosalicylic acid (4%). The mixture was centrifuged at 2500× *g* for 15 min. Then, 500 mL of supernatant was mixed with 500 μL of DTNB 10 mM. The absorbance was measured at 412 nm, and the concentration of GSH was expressed as mg/g of tissue.

#### 3.7.6. Histopathological Study

Histological examination was used to evaluate rat skin tissue. Skin fragments were fixed for 48 h in a formalin solution of 10%, dehydrated in an alcohol solution at an increasing concentration (70–100%), and impregnated with paraffin (56 to 57 °C). The paraffin blocks obtained were cut using a microtome colored with hematoxylin–eosin (HE) coloration and examined with an optic microscope. The inflammation damage scoring was as follows: mild, <10%; moderate, 10–50%; and marked, >50%.

#### 3.7.7. MN Assay in the Peripheral Blood

Fresh blood samples were treated with Ficoll 400 (Sigma reference: F4375-100G). The obtained samples were adjusted to 1 mL with phosphate-buffered saline solution (PBS) pH = 7.4. Then, they were centrifuged at 2500× *g* for 20 min. WBCs were extracted in the middle of the Ficoll gradient. Ficoll-extracted leukocytes were used to prepare the microscopic blade. Briefly, 50 μL of the extract was placed on microscopic glass slides. After drying, slides were treated with 5 mL of Carnoy solution successively for 5 and 10 min. Then, slides were dried and incubated in 3 mL of staining acridine orange solution (acridine orange/citric acid/Na_2_HPO_4_, H_2_O 4:16:1 *v*/*v*/*v*) for 10 min in the dark and washed twice for 5 min with distilled water. Finally, the slides were covered with a lamella in the dark, and the morphology of the cell nuclei was observed using a fluorescence microscope at an excitation wavelength of 520–560 nm.

### 3.8. Evaluation of Angiogenic Activity Using Chorioallantoic Membrane Assay

The PCT’s anti-angiogenic efficacy was evaluated according to Song et al. [95] with a slight modification. Briefly, hatching eggs were obtained on day 1 from the Eurafric Poussins company, Had Soualem, Maroc, Morocco, incubated until day 4 at 37.5 °C with 55% relative humidity, and rotated twice daily. On day 4, eggs were examined using an electric bulb to confirm the embryo’s development. At this stage, un-embryonated and dead embryos were removed. In a laminar air flow unit, the viable eggs were cleaned with ethanol (70%), and the albumin was aseptically extracted by drilling a hole in the pointed end of the egg; then using forceps, with great care, the shell’s membrane was separated from the shell to grant access to the CAM. The first group of eggs was used as a control, and 50 μL of distilled water was lightly brushed across the CAM containers. The other groups of eggs were treated with choriogonadotropin 0.5 μg/g egg, diclofenac 5 μg/g of egg, PCT 25 ug/g of egg, and PCT 50 ug/g of egg. After application of the treatment, eggs were closed with sterilized parafilm and re-incubated at 37 °C for 3 days. On day 7, photographs of blood vessels were taken using a research photo stereomicroscope (Steroblue EUROMEX Zeiss, Hamburg, Germany). The photomicrographs obtained from the CAM vessels were analyzed using the ImageJ (version 1.53) and AngioTool software (version v 0.6a).

The vascular network’s topography was measured using the software ImageJ. The images were converted to grayscale, and in sequence, they were “inverted” from the plugin “edit”. Next, the background was extracted from the images (a value of 50 pixels was fixed) in the “process” plugin. The photomicrographs were saved and opened using the Angio Tool software. The network topology was shown in a typical picture, formed by vessel area, vessel length, number of junctions, and lacunarity (gaps between vessels).

Score values for the anti-angiogenic effect on the CAM model were developed according to the method used by Bürgermeister et al. [75] to classify the severity of the angiogenic effect (Table 7). A score of 0 indicates normal capillary development, and 2 indicates an anti-angiogenic solid impact with a sizeable capillary-free area.

### 3.9. Statistical Analysis

Statistical significance was assessed with Graph Pad Prism version 9.0. Values were expressed as mean ± SEM. The Student’s *t*-test was used to determine the comparison between groups. At a *p*-value of 0.05, the difference in values between the control and treatment groups was deemed significant.

## 4. Conclusions

In this study, we have performed the extraction and biological characterization of polysaccharides isolated from green algae *C. tomentosum* for the first time. The results showed that PCT presented a powerful antioxidant, anti-angiogenic, and anti-inflammatory activity in a clear dose–response manner. PCT induces a reduction in the severity of oxidative stress, a positive regulation of antioxidant levels (SOD, GPx, GSH), DNA protection, and hematological regulation. Meanwhile, the anti-angiogenic capacity of PCT was demonstrated using the CAM model. Therefore, the biological and pharmacological characteristics of polysaccharides from *C. tomentosum* suggest their potential against inflammatory diseases. The druggability and pharmacokinetic properties of the PCT building blocks explain the assessed antioxidant and anti-angiogenic effects, which are the cause and/or the consequence of the promising anti-inflammatory potential, as evaluated in a murine model of acute inflammation.

## Figures and Tables

**Table 1:** Chemical composition of PCT extracted from *C. tomentosum*.

	PCT
Yield (%)	15.22% ± 0.05
Total sugar (%)	53.84% ± 0.19
Protein content (%)	2.05% ± 0.13
Sulfated groups (%)	5.71%± 0.07
Energy value	196.00 Kcal

**Table 2:** Druggability and pharmacokinetics of the algal-identified monosaccharides based on the physicochemical and ADMET properties.

Entry	Arabinose (1)	Fructose (2)	Galacturonic Acid (3)	Glucuronic Acid (4)	Rhamnose (5)	Xylose (6)
	Lipophilicity and physicochemical properties					
TPSA (Å^2^)	90.15	110.38	135.29	135.29	90.15	90.15
Log *P*o/w (iLOGP)	0.39	0.52	−0.68	−0.04	0.85	−0.39
Consensus Log *P*o/w	−1.85	−2.04	−2.30	−2.17	−1.42	−2.00
Log S (ESOL) solubility	1.13	0.90	0.91	0.91	0.46	1.13
	Pharmacokinetics					
GI absorption	Low	Low	Low	Low	High	Low
BBB permeant	No	No	No	No	No	No
P-gp substrate	No	No	No	No	Yes	No
CYP1A2	No	No	No	No	No	No
CYP2C19	No	No	No	No	No	No
CYP2C9	No	No	No	No	No	No
CYP2D6	No	No	No	No	No	No
CYP3A4	No	No	No	No	No	No

(1) Arabinose; (2) fructose; (3) galacturonic acid; (4) glucuronic acid; (5) rhamnose; (6) xylose.

**Table 4:** Effect of treatment with CARR, PCT, and/or DICL on hematological parameters.

Parameters	Control	CARR	CARR + PCT	CARR + DICL
WBC (10^3^/μL)	3.65 ± 0.55	14.26 ± 0.43 ^a^ ***	9.55 ± 0.97 ^b^ ***	9.80 ± 0.71 ^b^ ***
RBC (10^6^/μL)	9.71 ± 0.39	7.78 ± 0.57 ^a^ ***	8.53 ± 0.59 ^b^ ***	8.78 ± 0.43 ^b^ ***
PLT (10^3^/mm^3^)	960.66 ± 26.27	1105 ± 59.24 ^a^ ***	935.33 ± 21.08 ^b^ ***	986.33 ± 12.07 ^b^ ***
Hb (g/dL)	12.25 ± 0.66	10.80 ± 0.59 ^a^ ***	13.36 ± 0.73 ^b^ ***	12.31 ± 0.59 ^b^ ***
Ht (%)	39.75 ±1.44	41.11 ±1.11 ^a^ *	39.98 ± 1.48	43.00 ± 0.78 ^b^ *
MCV (mm^3^/RBC)	52.67 ± 1.35	51.23 ± 0.69 ^a^ *	51.79 ± 1.56	52.02 ± 1.79
MCH (pg/RBC)	18.76 ± 0.15	18.53 ± 0.85	18.54 ± 0.91	17.03 ± 0.05 ^b^ *

Control: normal rats injected with NaCl 0.9%; CARR: rats injected with carrageenan (1%); CARR + PCT: inflamed rats treated with 20 mg/kg; CARR + DICL: inflamed rats treated with diclofenac 25 mg/kg. Values are expressed as means ± SD for 6 animals in each group. a: compared to control; b: compared to CARR. * *p* < 0.05 and *** *p* < 0.001. WBC: white blood cell, RBC: red blood cell, PLT: platelet, Hb: hemoglobin, Ht: Hematocrit, MCV: mean corpuscular volume, MCH: mean corpuscular hemoglobin.

**Table 5:** Levels of serum proteins in control rats and rats treated with CARR, PCT, and/or DICL.

	Control	CARR	CARR + PCT	CARR + DICL
Total protein (g/dL)	56.46 ± 0.64	64.00 ± 0.10 ^a^ ***	57.05 ± 0.05 ^b^ ***	58.50 ± 0.50 ^b^ ***
Albumin (g/dL)	18.35 ± 0.35	17.30 ± 0.30 ^a^ ***	18.20 ± 0.40 ^b^ ***	18.51 ± 0.48 ^b^ ***
Alpha 1 globulins (g/dL)	7.36 ± 0.16	10.30 ± 0.30 ^a^ ***	7.50 ± 0.1 ^a^ * ^b^ ***	7.90 ± 0.01 ^b^ ***
Alpha 2 globulins (g/dL)	11.70 ± 0.10	12.55 ± 0.25 ^a^ ***	11.35 ± 0.34 ^b^ ***	11.32 ± 0.11 ^b^ ***
Beta 1 globulins (g/dL)	6.55 ± 0.35	7.30 ± 0.20 ^a^ ***	6.31 ± 0.29 ^b^ ***	6.20 ± 0.17 ^b^ ***
Beta 2 globulins (g/dL)	3.25 ± 0.25	4.45 ± 0.45 ^a^ ***	3.56 ± 0.08 ^b^ ***	3.90 ± 0.14 ^b^ **
Gamma globulins (g/dL)	7.06 ± 0.04	7.41 ± 0.08 ^a^ ***	7.08 ± 0.04 ^b^ ***	7.04 ± 0.04

Control: normal rats injected with NaCl 0.9%; CARR: rats injected with carrageenan 1%; CARR + PCT: inflamed rats treated with 20 mg/kg; CARR + DICL: inflamed rats treated with diclofenac 25 mg/kg. Values represent means ± SEM (n = 6) in each group. a: compared to control; b: compared to CARR. * *p* < 0.05, ** *p* < 0.01, and *** *p* < 0.001.

**Table 6:** Effect of CARR associated or not with PCT and DICL on MDA, AOPP, GPx, SOD, and GSH levels in rat paw skin and erythrocytes.

Parameters	Control	CARR	CARR + PCT	CARR + DICL
Paw skin				
MDA	18.75 ± 0.22	23.22 ± 0.13 ^a^ ***	18.53 ± 0.99 ^b^ ***	17.17 ± 0.13 ^b^ ***
AOPP	0.58 ± 0.04	0.75 ± 0.03 ^a^ ***	0.56 ± 0.07 ^b^ ***	0.56 ± 0.08 ^b^ ***
SOD	19.63 ± 1.12	13.97 ± 1.15 ^a^ ***	18.40 ± 1.28 ^b^ **	18.26 ± 0.79 ^b^ ***
GPx	10.49 ± 0.95	6.64 ± 0.99 ^a^ **	9.92 ± 0.56 ^b^ ***	10.83 ± 0.67 ^b^ ***
GSH	137.76 ± 0.98	147.11 ± 0.40 ^a^ ***	135.13 ± 0.53 ^b^ ***	138.78 ± 0.36 ^b^ ***
Erythrocytes				
MDA	80.83 ± 0.71	107.02 ±3.11 ^a^ **	74.16 ± 2.94 ^b^ **	85.71 ± 2.96 ^b^ **
AOPP	1.67 ± 0.07	3.32 ± 0.17 ^a^ ***	1.74 ± 0.08 ^b^ ***	1.91 ± 0.07 ^b^ ***
SOD	26.96 ± 1.33	14.15 ± 1.23 ^a^ ***	25.19± 1.35 ^b^ ***	26.98± 2.38 ^b^ ***
GPx	3.23 ± 0.10	2.26 ± 0.15 ^a^ ***	3.02 ± 0.39 ^b^ ***	3.38 ± 0.12 ^b^ ***
GSH	125.23 ± 2.34	137.98 ± 2.88 ^a^ ***	114.08 ± 2.41 ^b^ ***	117.02± 4.74 ^b^ ***

Control: normal rats injected with NaCl 0.9%; CARR: rats injected with carrageenan 1%; CARR + PCT: inflamed rats treated with 20 mg/kg; CARR + DICL: inflamed rats treated with diclofenac 25 mg/kg. Values are expressed as means ± SD for 6 animals in each group. a: compared to control; b: compared to CARR. ** *p* < 0.01, and *** *p* < 0.001. MDA: nmoles of MDA/mg protein; AOPP: μmoles/mg of protein; GSH: mg/g tissue; GPx: nmoles of GSH/min/mg protein; SOD: U/mg protein.

**Table 7:** Score values for the anti-angiogenic effect on the CAM model [75].

Score Value	Potential Effect	Observed Effect
0	No effect	
0.5	Very weak effect	No capillary-free area Area with reduced capillary density around the pellet (not larger than the area of the pellet)
1	Weak–medium effect	Small capillary-free area or area with significantly reduced capillary density (effect not larger than double the size of the pellet)
2	Strong effect	Capillary-free area around the pellet (at least double the size of the pellet)

## Data Availability

Data is contained within the article.

## References

[1] Lin Z., Zhang Q., Luo W. (2016). Angiogenesis Inhibitors as Therapeutic Agents in Cancer: Challenges and Future Directions. Eur. J. Pharmacol..

[2] Folkman J. (1971). Tumor Angiogenesis: Therapeutic Implications. N. Engl. J. Med..

[3] Prado M.R.M., Boller C., Zibetti R.G.M., de Souza D., Pedroso L.L., Soccol C.R. (2016). Anti-Inflammatory and Angiogenic Activity of Polysaccharide Extract Obtained from *Tibetan kefir*. Microvasc. Res..

[4] Ferrara N., Kerbel R.S. (2005). Angiogenesis as a Therapeutic Target. Nature.

[5] Adams R.H., Alitalo K. (2007). Molecular Regulation of Angiogenesis and Lymphangiogenesis. Nat. Rev. Mol. Cell Biol..

[6] Jayson G.C., Kerbel R., Ellis L.M., Harris A.L. (2016). Antiangiogenic Therapy in Oncology: Current Status and Future Directions. Lancet.

[7] Mantovani A., Allavena P., Sica A., Balkwill F. (2008). Cancer-Related Inflammation. Nature.

[8] Srinivasan K., Muruganandan S., Lal J., Chandra S., Tandan S.K., Ravi Prakash V. (2001). Evaluation of Anti-Inflammatory Activity of *Pongamia pinnata* Leaves in Rats. J. Ethnopharmacol..

[9] Gupta S.C., Kunnumakkara A.B., Aggarwal S., Aggarwal B.B. (2018). Inflammation, a Double-Edge Sword for Cancer and Other Age-Related Diseases. Front. Immunol..

[10] Zhang D., Tan L.-H., Feng Y.-J., Yao L., Yan X.-W., Cao W.-G. (2021). Evaluation of Antioxidant, Anti-Inflammatory Activity and Identification of Active Compounds of *Humulus scandens*. S. Afr. J. Bot..

[11] Osifo M., Ihim S.A., Ani N., Nworu C.S., Akah P. (2022). Wound Healing and Anti-Inflammatory Activities of *Ceiba pentendra* (l.) Gaertn. Pharmacol. Res.-Mod. Chin. Med..

[12] Wang Y., Guo X., Huang C., Shi C., Xiang X. (2024). Biomedical Potency and Mechanisms of Marine Polysaccharides and Oligosaccharides: A Review. Int. J. Biol. Macromol..

[13] Ale M.T., Maruyama H., Tamauchi H., Mikkelsen J.D., Meyer A.S. (2011). Fucoidan from *Sargassum* sp. and *Fucus vesiculosus* Reduces Cell Viability of Lung Carcinoma and Melanoma Cells in Vitro and Activates Natural Killer Cells in Mice in vivo. Int. J. Biol. Macromol..

[14] Cumashi A., Ushakova N.A., Preobrazhenskaya M.E., D’Incecco A., Piccoli A., Totani L., Tinari N., Morozevich G.E., Berman A.E., Bilan M.I. (2007). A Comparative Study of the Anti-Inflammatory, Anticoagulant, Antiangiogenic, and Antiadhesive Activities of Nine Different Fucoidans from Brown Seaweeds. Glycobiology.

[15] Costa L.E.C., Brito T.V., Damasceno R.O.S., Sousa W.M., Barros F.C.N., Sombra V.G., Júnior J.S.C., Magalhães D.A., Souza M.H.L.P., Medeiros J.-V.R. (2020). Chemical Structure, Anti-Inflammatory and Antinociceptive Activities of a Sulfated Polysaccharide from Gracilaria Intermedia Algae. Int. J. Biol. Macromol..

[16] de Souza L.A.R., Dore C.M.P.G., Castro A.J.G., de Azevedo T.C.G., deOliveira M.T.B., de Fátima M.F.V., Benevides N.M.B., Leite E.L. (2012). Galactans from the Red Seaweed *Amansia multifida* and Their Effects on Inflammation, Angiogenesis, Coagulation and Cell Viability. Biomed. Prev. Nutr..

[17] Sousa S.G., Oliveira L.A., de Aguiar Magalhães D., de Brito T.V., Batista J.A., Pereira C.M.C., de Souza Costa M., Mazulo J.C.R., de Carvalho Filgueiras M., Vasconselos D.F.P. (2018). Chemical Structure and Anti-Inflammatory Effect of Polysaccharide Extracted from *Morinda citrifolia* Linn (Noni). Carbohydr. Polym..

[18] Wijesekara I., Pangestuti R., Kim S.-K. (2011). Biological Activities and Potential Health Benefits of Sulfated Polysaccharides Derived from Marine Algae. Carbohydr. Polym..

[19] Wei H., Shi Y., Yuan Z., Huang Z., Cai F., Zhu J., Zhang W., Li J., Xiong Q., Wang Y. (2021). Isolation, Identification, and Anti-Inflammatory Activity of Polysaccharides of *Typha angustifolia*. Biomacromolecules.

[20] Albuquerque I.R.L., Cordeiro S.L., Gomes D.L., Dreyfuss J.L., Filgueira L.G.A., Leite E.L., Nader H.B., Rocha H.A.O. (2013). Evaluation of Anti-Nociceptive and Anti-Inflammatory Activities of a Heterofucan from *Dictyota menstrualis*. Mar. Drugs.

[21] Ananthi S., Raghavendran H.R.B., Sunil A.G., Gayathri V., Ramakrishnan G., Vasanthi H.R. (2010). In Vitro Antioxidant and in Vivo Anti-Inflammatory Potential of Crude Polysaccharide from *Turbinaria ornata* (Marine Brown Alga). Food Chem. Toxicol. Int. J. Public Br. Ind. Biol. Res. Assoc..

[22] Carneiro J.G., Rodrigues J.A.G., de Sousa Oliveira Vanderlei E., Souza R.B., Quinderé A.L.G., Coura C.O., de Araújo I.W.F., Chaves H.V., Bezerra M.M., Benevides N.M.B. (2014). Peripheral Antinociception and Anti-Inflammatory Effects of Sulphated Polysaccharides from the Alga *Caulerpa mexicana*. Basic Clin. Pharmacol. Toxicol..

[23] Fernández P.V., Arata P.X., Ciancia M. (2014). Polysaccharides from *Codium* Species. Advances in Botanical Research.

[24] Wang L., Wang X., Wu H., Liu R. (2014). Overview on Biological Activities and Molecular Characteristics of Sulfated Polysaccharides from Marine Green Algae in Recent Years. Mar. Drugs.

[25] Celikler S., Vatan O., Yildiz G., Bilaloglu R. (2009). Evaluation of Anti-Oxidative, Genotoxic and Antigenotoxic Potency of *Codium tomentosum* Stackhouse Ethanolic Extract in Human Lymphocytes in Vitro. Food Chem. Toxicol..

[26] Tabarsa M., Karnjanapratum S., Cho M., Kim J.-K., You S. (2013). Molecular Characteristics and Biological Activities of Anionic Macromolecules from *Codium fragile*. Int. J. Biol. Macromol..

[27] Fernández P.V., Raffo M.P., Alberghina J., Ciancia M. (2015). Polysaccharides from the Green Seaweed *Codium decorticatum*. Structure and Cell Wall Distribution. Carbohydr. Polym..

[28] Li N., Mao W., Yan M., Liu X., Xia Z., Wang S., Xiao B., Chen C., Zhang L., Cao S. (2015). Structural Characterization and Anticoagulant Activity of a Sulfated Polysaccharide from the Green Alga *Codium divaricatum*. Carbohydr. Polym..

[29] Hamzaoui A., Ghariani M., Sellem I., Hamdi M., Feki A., Jaballi I., Nasri M., Amara I.B. (2020). Extraction, Characterization and Biological Properties of Polysaccharide Derived from Green Seaweed “*Chaetomorpha linum*” and Its Potential Application in Tunisian Beef Sausages. Int. J. Biol. Macromol..

[30] Jaballi I., Sallem I., Feki A., Cherif B., Kallel C., Boudawara O., Jamoussi K., Mellouli L., Nasri M., Amara I.B. (2019). Polysaccharide from a Tunisian Red Seaweed *Chondrus canaliculatus*: Structural Characteristics, Antioxidant Activity and In Vivo Hemato-Nephroprotective Properties on Maneb Induced Toxicity. Int. J. Biol. Macromol..

[31] Wang J., Zhang Q., Zhang Z., Li Z. (2008). Antioxidant Activity of Sulfated Polysaccharide Fractions Extracted from *Laminaria japonica*. Int. J. Biol. Macromol..

[32] Tsubaki S., Oono K., Hiraoka M., Onda A., Mitani T. (2016). Microwave-Assisted Hydrothermal Extraction of Sulfated Polysaccharides from *Ulva* spp. and *Monostroma latissimum*. Food Chem..

[33] Kravchenko A.O., Byankina Barabanova A.O., Glazunov V.P., Yakovleva I.M., Yermak I.M. (2018). Seasonal Variations in a Polysaccharide Composition of Far Eastern Red Seaweed *Ahnfeltiopsis flabelliformis* (Phyllophoraceae). J. Appl. Phycol..

[34] Manikandan R., Parimalanandhini D., Mahalakshmi K., Beulaja M., Arumugam M., Janarthanan S., Palanisamy S., You S., Prabhu N.M. (2020). Studies on Isolation, Characterization of Fucoidan from Brown Algae *Turbinaria decurrens* and Evaluation of It’s In Vivo and In Vitro Anti-Inflammatory Activities. Int. J. Biol. Macromol..

[35] Kolsi R.B.A., Jardak N., Hajkacem F., Chaaben R., El Feki A., Rebai T., Jamoussi K., Fki L., Belghith H., Belghith K. (2017). Anti-Obesity Effect and Protection of Liver-Kidney Functions by *Codium fragile* Sulphated Polysaccharide on High Fat Diet Induced Obese Rats. Int. J. Biol. Macromol..

[36] Figueroa F.A., Abdala-Díaz R.T., Pérez C., Casas-Arrojo V., Nesic A., Tapia C., Durán C., Valdes O., Parra C., Bravo-Arrepol G. (2022). Sulfated Polysaccharide Extracted from the Green Algae *Codium bernabei*: Physicochemical Characterization and Antioxidant, Anticoagulant and Antitumor Activity. Mar. Drugs.

[37] Rioux L.-E., Turgeon S.L., Beaulieu M. (2009). Effect of Season on the Composition of Bioactive Polysaccharides from the Brown Seaweed *Saccharina longicruris*. Phytochemistry.

[38] Gao X., Qu H., Shan S., Song C., Baranenko D., Li Y., Lu W. (2020). A Novel Polysaccharide Isolated from *Ulva pertusa*: Structure and Physicochemical Property. Carbohydr. Polym..

[39] Zammel N., Saeed M., Bouali N., Elkahoui S., Alam J.M., Rebai T., Kausar M.A., Adnan M., Siddiqui A.J., Badraoui R. (2021). Antioxidant and Anti-Inflammatory Effects of *Zingiber officinale roscoe* and *Allium subhirsutum*: In Silico, Biochemical and Histological Study. Foods.

[40] Badraoui R., Saeed M., Bouali N., Hamadou W.S., Elkahoui S., Alam M.J., Siddiqui A.J., Adnan M., Saoudi M., Rebai T. (2022). Expression Profiling of Selected Immune Genes and Trabecular Microarchitecture in Breast Cancer Skeletal Metastases Model: Effect of α-Tocopherol Acetate Supplementation. Calcif. Tissue Int..

[41] Bédoui I., Nasr H., Ksouda K., Ayadi W., Louati N., Chamkha M., Choura S., Gargouri J., Hammami S., Affes H. (2023). Phytochemical Composition, Bioavailability and Pharmacokinetics of *Scorzonera undulata* Methanolic Extracts: Antioxidant, Anticancer, and Apoptotic Effects on MCF7 Cells. Pharmacogn. Mag..

[42] Rahmouni F., Badraoui R., Nasr H., Bardakci F., Elkahoui S., Siddiqui A., Saeed M., Mejdi S., Saoudi M., Rebai T. (2022). Pharmacokinetics and Therapeutic Potential of *Teucrium polium* against Liver Damage Associated Hepatotoxicity and Oxidative Injury in Rats: Computational, Biochemical and Histological Studies. Life.

[43] Zammel N., Jedli O., Rebai T., Hamadou W., Elkahoui S., Jamal A., Alam M., Adnan M., Siddiqui A., Alreshidi M. (2022). Kidney Injury and Oxidative Damage Alleviation by *Zingiber officinale*: Pharmacokinetics and Protective Approach in a Combined Murine Model of Osteoporosis. 3 Biotech.

[44] Physico-Chemical Properties, Pharmacokinetics, Molecular Docking and In-Vitro Pharmacological Study of a Cobalt (II) Complex Based on 2-Aminopyridine—Mhadhbi—2022—ChemistrySelect—Wiley Online Library. https://chemistry-europe.onlinelibrary.wiley.com/doi/abs/10.1002/slct.202103592.

[45] Jedli O., Ben-Nasr H., Zammel N., Rebai T., Saoudi M., Elkahoui S., Jamal A., Siddiqui A.J., Sulieman A.E., Alreshidi M.M. (2022). Attenuation of Ovalbumin-Induced Inflammation and Lung Oxidative Injury in Asthmatic Rats by *Zingiber officinale* Extract: Combined in Silico and in Vivo Study on Antioxidant Potential, STAT6 and TNF-α Pathways. 3 Biotech.

[46] Jebahi S., Ben Salah G., Jarray S., Naffati M., Ahmad M.A., Brahmi F., Saeed M., Siddiqui A.J., Abdelmajid K., Badraoui R. (2022). Chitosan-Based Gastric Dressing Materials Loaded with Pomegranate Peel as Bioactive Agents: Pharmacokinetics and Effects on Experimentally Induced Gastric Ulcers in Rabbits. Metabolites.

[47] Badraoui R., Adnan M., Bardakci F., Alreshidi M. (2021). Chloroquine and Hydroxychloroquine Interact Differently with ACE2 Domains Reported to Bind with the Coronavirus Spike Protein: Mediation by ACE2 Polymorphism. Molecules.

[48] Tian H., Liu H., Song W., Zhu L., Yin X. (2021). Polysaccharide from *Caulerpa lentillifera*: Extraction Optimization with Response Surface Methodology, Structure and Antioxidant Activities. Nat. Prod. Res..

[49] Yuan Y., Xu X., Jing C., Zou P., Zhang C., Li Y. (2018). Microwave Assisted Hydrothermal Extraction of Polysaccharides from *Ulva prolifera*: Functional Properties and Bioactivities. Carbohydr. Polym..

[50] Yarley O.P.N., Kojo A.B., Zhou C., Yu X., Gideon A., Kwadwo H.H., Richard O. (2021). Reviews on Mechanisms of in Vitro Antioxidant, Antibacterial and Anticancer Activities of Water-Soluble Plant Polysaccharides. Int. J. Biol. Macromol..

[51] Raposo M., De Morais R., Bernardo de Morais A. (2013). Bioactivity and Applications of Sulphated Polysaccharides from Marine Microalgae. Mar. Drugs.

[52] Ouahid E.A., Mohamed R., Soufiane F., Inamuddin, Ahamed M.I., Boddula R., Altalhi T. (2021). Green Seaweed Polysaccharides Inventory of Nador Lagoon in North East Morocco. Polysaccharides.

[53] Yuan Q., Li H., Wei Z., Lv K., Gao C., Liu Y., Zhao L. (2020). Isolation, Structures and Biological Activities of Polysaccharides from Chlorella: A Review. Int. J. Biol. Macromol..

[54] Feki A., Cherif B., Sellem I., Naifar M., Ben Amar I., Ben Azaza Y., Kallel R., Hariz L., Zeghal S., Makni Ayadi F. (2023). Biomedical Applications of Polysaccharide Derived from *Tetrasporophyte tufts* of *Asparagopsis armata* (*Falkenbergia rufolanosa*): Focus on Antioxidant, Anti-Inflammatory, Anti-Coagulant and Hepato-Protective Activities. Algal Res..

[55] Kraiem M., Ben Hamouda S., Eleroui M., Ajala M., Feki A., Dghim A., Boujhoud Z., Bouhamed M., Badraoui R., Pujo J.M. (2024). Anti-Inflammatory and Immunomodulatory Properties of a Crude Polysaccharide Derived from Green Seaweed *Halimeda tuna*: Computational and Experimental Evidences. Mar. Drugs.

[56] Hao H., Han Y., Yang L., Hu L., Duan X., Yang X., Huang R. (2019). Structural Characterization and Immunostimulatory Activity of a Novel Polysaccharide from Green Alga *Caulerpa racemosa* var *peltata*. Int. J. Biol. Macromol..

[57] Varela M.L., Mogildea M., Moreno I., Lopes A. (2018). Acute Inflammation and Metabolism. Inflammation.

[58] Winter C.A., Risley E.A., Nuss G.W. (1962). Carrageenin-Induced Edema in Hind Paw of the Rat as an Assay for Antiinflammatory Drugs. Exp. Biol. Med..

[59] Posadas I., Bucci M., Roviezzo F., Rossi A., Parente L., Sautebin L., Cirino G. (2004). Carrageenan-Induced Mouse Paw Oedema Is Biphasic, Age-Weight Dependent and Displays Differential Nitric Oxide Cyclooxygenase-2 Expression. Br. J. Pharmacol..

[60] Xu S.-Y., Huang X., Cheong K.-L. (2017). Recent Advances in Marine Algae Polysaccharides: Isolation, Structure, and Activities. Mar. Drugs.

[61] de Jesus Raposo M.F., de Morais A.M.B., de Morais R.M.S.C. (2015). Marine Polysaccharides from Algae with Potential Biomedical Applications. Mar. Drugs.

[62] Bhardwaj M., Mani S., Malarvizhi R., Sali V.K., Vasanthi H.R. (2021). Immunomodulatory Activity of Brown Algae *Turbinaria ornata* Derived Sulfated Polysaccharide on LPS Induced Systemic Inflammation. Phytomedicine.

[63] Bouali N., Hamadou W.S., Badraoui R., Lajimi R.H., Hamdi A., Alreshidi M., Adnan M., Soua Z., Siddiqui A.J., Noumi E. (2022). Phytochemical Composition, Antioxidant, and Anticancer Activities of Sidr Honey: In Vitro and In Silico Computational Investigation. Life.

[64] Germolec D.R., Shipkowski K.A., Frawley R.P., Evans E., DeWitt J.C., Rockwell C.E., Bowman C.C. (2018). Markers of Inflammation. Immunotoxicity Testing.

[65] Mzid M., Ben Khedir S., Bardaa S., Sahnoun Z., Rebai T. (2017). Chemical Composition, Phytochemical Constituents, Antioxidant and Anti-Inflammatory Activities of *Urtica urens* L. Leaves. Arch. Physiol. Biochem..

[66] Egan C.G., Lockhart J.C., Ferrell W.R., Day S.M., McLean J.S. (2002). Pathophysiological Basis of Acute Inflammatory Hyperaemia in the Rat Knee: Roles of Cyclo-Oxygenase-1 and -2. J. Physiol..

[67] Geboes K. (1994). From Inflammation to Lesion. Acta Gastro-Enterol. Belg..

[68] Feki A., Jaballi I., Cherif B., Ktari N., Naifar M., Makni Ayadi F., Kallel R., Boudawara O., Kallel C., Nasri M. (2019). Therapeutic Potential of Polysaccharide Extracted from Fenugreek Seeds against Thiamethoxam-Induced Hepatotoxicity and Genotoxicity in Wistar Adult Rats. Toxicol. Mech. Methods.

[69] di Filippo P., Sousa W., Fujimoto T., Mota F., Alves A. (2020). Acute Phase Proteins Response and Their Clinical Application in Veterinary Medicine. Veterinária Notícias.

[70] Tothova C., Nagy O., Kovac G. (2014). Acute Phase Proteins and Their Use in the Diagnosis of Diseases in Ruminants: A Review. Veterinární Medicína.

[71] Holanda B.F., Freitas de Araujo D., da Silva J.N.R., Pereira M.G., de Freitas Pires A., Assreuy A.M. (2021). Polysaccaride-Rich Extract of Caesalpina Ferrea Stem Barks Attenuates Mice Acute Inflammation Induced by Zymosan: Oxidative Stress Modulation. J. Ethnopharmacol..

[72] Jaballi I., Saad H.B., Bkhairia I., Cherif B., Kallel C., Boudawara O., Droguet M., Magné C., Hakim A., Amara I.B. (2018). Cytoprotective Effects of the Red Marine Alga *Chondrus canaliculatus* against Maneb-Induced Hematotoxicity and Bone Oxidative Damages in Adult Rats. Biol. Trace Elem. Res..

[73] Ben Amara I., Ben Saad H., Cherif B., Elwej A., Lassoued S., Kallel C., Zeghal N. (2014). Methyl-Thiophanate Increases Reactive Oxygen Species Production and Induces Genotoxicity in Rat Peripheral Blood. Toxicol. Mech. Methods.

[74] Souza R.B., Frota A.F., Silva J., Alves C., Neugebauer A.Z., Pinteus S., Rodrigues J.A.G., Cordeiro E.M.S., de Almeida R.R., Pedrosa R. (2018). In Vitro Activities of Kappa-Carrageenan Isolated from Red Marine Alga *Hypnea musciformis*: Antimicrobial, Anticancer and Neuroprotective Potential. Int. J. Biol. Macromol..

[75] Bürgermeister J., Paper D.H., Vogl H., Linhardt R.J., Franz G. (2002). LaPSvS1, a (1→3)-β-Galactan Sulfate and Its Effect on Angiogenesis in vivo and in vitro. Carbohydr. Res..

[76] Zheng C., Dong Q., Du Z., Wang P., Ding K. (2015). Structural Elucidation of a Polysaccharide from *Chrysanthemum morifolium* Flowers with Anti-Angiogenic Activity. Int. J. Biol. Macromol..

[77] Ding J., Jia W., Cui Y., Jin J., Zhang Y., Xu L., Liu Y. (2020). Anti-Angiogenic Effect of a Chemically Sulfated Polysaccharide from *Phellinus ribis* by Inhibiting VEGF/VEGFR Pathway. Int. J. Biol. Macromol..

[78] Liu F., Wang J., Chang A.K., Liu B., Yang L., Li Q., Wang P., Zou X. (2012). Fucoidan Extract Derived from *Undaria pinnatifida* Inhibits Angiogenesis by Human Umbilical Vein Endothelial Cells. Phytomedicine.

[79] Xiong Q., Hao H., He L., Jing Y., Xu T., Chen J., Zhang H., Hu T., Zhang Q., Yang X. (2017). Anti-Inflammatory and Anti-Angiogenic Activities of a Purified Polysaccharide from Flesh of *Cipangopaludina chinensis*. Carbohydr. Polym..

[80] Liu J., Willför S., Xu C. (2015). A Review of Bioactive Plant Polysaccharides: Biological Activities, Functionalization, and Biomedical Applications. Bioact. Carbohydr. Diet. Fibre.

[81] DuBois M., Gilles K.A., Hamilton J.K., Rebers P.T., Smith F. (1956). Colorimetric Method for Determination of Sugars and Related Substances. Anal. Chem..

[82] Lloyd A.G., Dodgson K.S., Price R.G. (1963). Comparative studies on cartilage aminopolysaccharide sulphates I. Polysaccharides from shark, skate, dogfish and fin whale. Biochim. Biophys. Acta (BBA).

[83] Lowry O.H., Rosebrough N.J., Farr A.L., Randall R.J. (1951). Protein measurement with the Folin phenol reagent. J. Biol. Chem..

[84] He R., Zhao Y., Zhao R., Sun P. (2015). Antioxidant and Antitumor Activities in vitro of Polysaccharides from *E. sipunculoides*. Int. J. Biol. Macromol..

[85] Bayar N., Kriaa M., Kammoun R. (2016). Extraction and Characterization of Three Polysaccharides Extracted from *Opuntia ficus* Indica Cladodes. Int. J. Biol. Macromol..

[86] Lopes-Lutz D., Alviano D.S., Alviano C.S., Kolodziejczyk P.P. (2008). Screening of Chemical Composition, Antimicrobial and Antioxidant Activities of Artemisia Essential Oils. Phytochemistry.

[87] Prieto P., Pineda M., Aguilar M. (1999). Spectrophotometric Quantitation of Antioxidant Capacity through the Formation of a Phosphomolybdenum Complex: Specific Application to the Determination of Vitamin E. Anal. Biochem..

[88] Yıldırım A., Mavi A., Kara A.A. (2001). Determination of Antioxidant and Antimicrobial Activities of *Rumex crispus* L. Extracts. J. Agric. Food Chem..

[89] Ijarotimi O.S., Adeoti O.A., Ariyo O. (2013). Comparative Study on Nutrient Composition, Phytochemical, and Functional Characteristics of Raw, Germinated, and Fermented *Moringa oleifera* Seed Flour. Food Sci. Nutr..

[90] Ohkawa H., Ohishi N., Yagi K. (1979). Assay for Lipid Peroxides in Animal Tissues by Thiobarbituric Acid Reaction. Anal. Biochem..

[91] Witko V., Nguyen A.T., Descamps-Latscha B. (1992). Microtiter Plate Assay for Phagocyte-Derived Taurine-Chloramines. J. Clin. Lab. Anal..

[92] Beauchamp C., Fridovich I. (1971). Superoxide Dismutase: Improved Assays and an Assay Applicable to Acrylamide Gels. Anal. Biochem..

[93] Flohé L., Günzler W.A. (1984). Assays of Glutathione Peroxidase. Methods Enzymol..

[94] Moron M., Depierre J., Mannervik B. (1979). Levels of Glutathione, Glutathione Reductase and Glutathione S-Transferase Activities in Rat Lung and Liver. Biochim. Biophys. Acta BBA-Gen. Subj..

[95] Song Y.S., Kim S.-H., Sa J.-H., Jin C., Lim C.-J., Park E.-H. (2004). Anti-Angiogenic and Inhibitory Activity on Inducible Nitric Oxide Production of the Mushroom *Ganoderma lucidum*. J. Ethnopharmacol..

